# Rational Design of Multifunctional Tacrine Derivatives as Candidates for the Treatment of Alzheimer and Parkinson Diseases

**DOI:** 10.1002/cmdc.70429

**Published:** 2026-08-10

**Authors:** Eugenia Dzib, Luis Felipe Hernández‐Ayala, Silvana Silva‐Aguirre, Annia Galano

**Affiliations:** ^1^ Departamento de Química División de Ciencias Básicas e Ingeniería Universidad Autónoma Metropolitana, Unidad Iztapalapa Ciudad de México México; ^2^ Departamento de Física Aplicada Centro de Investigación y de Estudios Avanzados, Unidad Mérida Mérida Yucatán México

**Keywords:** binding, computational, neurodegeneration, rational drug design, reactivity

## Abstract

Alzheimer disease (AD) and Parkinson disease (PD) are multifactorial neurodegenerative disorders for which there is currently no therapy that prevents or slows their progress. Some drugs used to treat AD are inhibitors of acetylcholinesterase (AChE) and antagonists of N‐methyl‐D‐aspartate receptor (NMDAr), while inhibitors of catechol‐*O*‐methyltransferase (COMT) and monoamine oxidase B (MAO‐B) are used for PD. Tacrine was the first FDA (Food and Drug Administration) approved drug against AD. Although later withdrawn due to hepatotoxicity, it remains a pivotal scaffold for drug development. Herein, 1295 tacrine derivatives, meant to enhance therapeutic efficacy and safety of the parent compound, were designed through the CADMA‐Chem protocol. The chemical space was screened using selection scores based on ADME properties, toxicity, and synthetic accessibility. Two derivatives with the best drug‐like behavior were chosen for further investigation. Acid‐base constants and reactivity descriptors were estimated for them. Our findings show that these derivatives are promising inhibitors of AChE, COMT, NMDAr, and MAO‐B. Therefore, according to in silico predictions they are expected to be beneficial for AD and PD. One of the compounds investigated here is the first reported tacrine‐derived compound with potential as COMT inhibitor.

## Graphical abstract

We present a set of multifunctional tacrine derivatives, designed with the CADMA‐Chem protocol, aimed for Alzheimer and Parkinson diseases. Of the tacrines designed, TAC‐674 and TAC‐678 are the best candidates to act as multifunctional antioxidants and neuroprotectors for Alzheimer and Parkinson diseases, respectively.

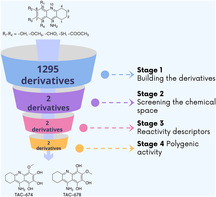

## 1 Introduction

Alzheimer disease (AD) and Parkinson disease (PD) are the most prevalent neurodegenerative disorders that currently affect humankind. In 2021, 40 million [1] and 11.8 million [2] people worldwide were living with AD and PD, respectively; and these numbers are projected to rise to 132 million [3] and 25.2 million [4] people, respectively, by 2050.

AD is characterized by progressive memory loss and cognitive impairment. AD patients exhibits accumulation of extracellular amyloid‐ß (Aß) plaques and intracellular tau neurofibrillary tangles (NFTs) [5, 6]. Other cause of AD is the dramatic decrease of the acetylcholine (ACh) levels due to a decline in ACh synthesis by choline acetyltransferase (ChAT) combined with an increase in ACh degradation by acetylcholinesterase (AChE) [6, 7, 8]. Besides, overstimulation of the N‐methyl‐D‐aspartate receptor (NMDAr) leads to excessive sodium and calcium ions, resulting in excitotoxic damage to the neurons [6, 9, 10, 11]. Finally, oxidative stress (OS), caused by an imbalance between the production of oxidizing species and their removal by the antioxidant defense system, significantly contributes to the progression of AD and is tightly linked to its aforesaid causes [6, 12, 13, 14, 15].

The key hallmarks of PD encompass the aggregation of α‐synuclein (α‐syn) as Lewy bodies [16, 17, 18] and the progressive loss of dopaminergic neurons in the *substantia nigra pars compacta* (SNpc) leading to a depletion of dopamine (DA) levels. This triggers the onset of the characteristic motor symptoms of PD such as tremor, bradykinesia, stiffness, and postural instability [16, 19]. DA is a neurotransmitter essential for motor control, cognition, reward, and modulation of emotions [20, 21]. Nevertheless, current evidence suggests that its oxidation induce oxidative damage in dopaminergic neurons leading to their degeneration and death [19]. DA oxidation can be spontaneous or mediated by metal ions or enzymes, primarily by monoamine oxidase B (MAO‐B) and catechol‐*O*‐methyltransferase (COMT) [16, 19, 20]. The latter also metabolizes levodopa (L‐DOPA) which is a DA precursor [20]. With aging the MAO‐B activity is increased in the human brain leading to a DA deficiency and a high production of H_2_O_2_ and toxic aldehydes [16, 22, 23, 24].

Two types of drugs are commonly used for treating AD: AChE inhibitors such as donepezil, rivastigmine, and galantamine for mild and moderate stages of AD [5, 6, 7, 15, 16], and the NMDA receptor antagonist memantine for moderate and severe stages of AD [5, 6, 15, 16]. The anti‐Aß monoclonal antibodies lecanemab [25, 26] and donanemab [26, 27] were approved by the FDA (Food and Drug Administration) in 2023 and 2024, respectively, for early‐stage AD. On the other hand, the most efficacious anti‐parkinsonian drug is L‐DOPA, but it has a short half‐life. Thus, it is frequently combined with COMT inhibitors such as opicapone, entacapone, and tolcapone (only this can cross blood brain barrier), and MAO‐B inhibitors such as rasagiline, selegiline, and safinamide. L‐DOPA and MAO‐B inhibitors are used when PD is at early stages, while COMT inhibitors are recommended for late stages [16, 23, 28, 29, 30]. However, except for lecanemab [25, 26] and donanemab [26, 27], these drugs only relieve the symptoms, they cannot slow down or inhibit the progression of AD and PD [31]. Furthermore, lecanemab [25, 26, 32] and donanemab [26, 27] can lead to serious adverse effects. Therefore, due to the multifactorial nature of AD and PD as well as the limitations of the currently used therapies to treat these diseases, it is urgent to design multitarget small drugs that can improve therapeutic efficacy and safety.

**SCHEME 1:**
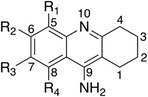
Tacrine (1,2,3,4‐tetrahydroacridin‐9‐amine, R_1_–R_4_ = H, TAC) and the substitution sites used in this work.

Tacrine (9‐amine‐1,2,3,4‐tetrahydroacridine, TAC, Scheme 1) was the pioneering drug approved by FDA, in 1993, to treat AD. It acts by inhibiting AChE to prevent ACh degradation and it was used in mild‐to‐moderate AD. However, it was discontinued in 2013 due to its hepatotoxicity [33, 34, 35, 36, 37, 38]. Evidence strongly suggests that the 7‐hidroxytacrine (7‐OH‐TAC) metabolite is the culprit of tacrine‐associated hepatotoxicity. The subsequent oxidation of 7‐OH‐TAC produces the reactive quinone methide that conjugates with intracellular proteins [39, 40]. After its ban, tacrine has been widely used as an important starting point for drug development. This is because of its strong binding to AChE [34, 41], synthetic accessibility [34], ligand efficiency, simple structure [33, 34], suitability for structural modification [34, 38], and low molecular weight [34, 42]. What is more, tacrine possess a quinoline moiety that is particularly relevant in medicinal chemistry and pharmaceutic industry [43, 44, 45]. Immense efforts have been focused on finding safer tacrine derivatives (mainly linked or merged tacrine‐based hybrids) [34, 35, 36, 37, 38, 41, 42, 46, 47, 48]. These derivatives have shown a significant inhibitory potential against the proteins AChE [34, 35, 36, 37, 38, 41, 42, 46, 47, 48], NMDA receptor [36, 38, 42, 48], and MAO‐B [36, 38, 41]. On the other hand, to our best knowledge, no tacrine derivatives capable of inhibiting COMT have been developed yet.

Inspired by the multi‐target approach, a systematic and rational search of tacrine derivatives with antioxidant and neuroprotective behavior for treating Alzheimer and Parkinson diseases was performed. This was carried out using a protocol known as CADMA‐Chem (Computer‐Assisted Design of Multifunctional Antioxidants based on chemical properties) which serves to identify viable compounds to treat polygenic diseases (i.e., multifactorial disorders) [49, 50]. To date, it has been used for designing neuroprotective compounds with promising results by our group [49, 50, 51, 52, 53, 54, 55] and other researchers [56, 57, 58, 59, 60, 61, 62, 63]. The derivatives were obtained by adding small substituents to the tacrine's saturated ring, avoiding the drawbacks related to the high molecular weight producing a suboptimal drug‐like behavior [64]. Drug‐like behavior, toxicity, manufacturability, antioxidant activity through electron and hydrogen atom donating capabilities, and polygenic protection by inhibiting the proteins AChE, NMDAr, COMT, and MAO‐B were all estimated. Based on these results, the most promising candidates were proposed for future research.

## 2 Computational Details

### 2.1 Building the Derivatives

Tacrine derivatives were built by inserting up to four functional groups (i.e., –OH, –OCH_3_, –CHO, –SH, and –COOCH_3_) into the unsaturated sites of tacrine (Scheme 1, TAC‐*n*, where *n* is the derivative number). These substituents were selected due to their ability to influence solubility, acid–base behavior, antiradical activity, and metal chelating capacity. The derivatives were designed with the *Smile‐it* program (version 1.0) [50, 65].

### 2.2 Screening the Chemical Space

The absorption, distribution, metabolism, and elimination (ADME) properties were predicted for all the derivatives with the *CADMA‐Py* [49, 50, 66] program. *CADMA‐Py* estimates the ADME properties of molecular weight (MW), water/octanol partition coefficient (logP), molar refractivity (MR), number of heavy atoms (XAt), number of H‐bond acceptors (HBA), number of H‐bond donors (HBD), rotatable bonds (RB), and topological polar surface area (PSA). These parameters are needed to investigate if the derivatives satisfy the rules of Lipinski [67], Ghose [68], Egan [69], Muegge [70], and Veber [71].

The toxicity (T) was evaluated by computing the oral rat 50% lethal dose (LD_50_), Ames mutagenicity (M), and developmental toxicity (DT). Additionally, the hepatotoxicity was estimated by calculating the drug‐induced liver injury (DILI) descriptor. To assess the reliability of the toxicity results several programs were used (Table S1). However, some of them were not suitable for this work (e.g., they offer only qualitative information for some toxicity parameters, the tacrine derivatives are out of their applicability domain, etc.). The programs selected to estimate the toxicity parameters are listed in Table 1. Consequently, three software combinations resulted in toxicity evaluation: (1) admetSAR3.0 for LD_50_ and M, and DeTox for DT; (2) admetSAR3.0 for LD_50_, ADMETlab3.0 for M, and DeTox for DT; and (3) admetSAR3.0 for LD_50_, ProTox3.0 for M, and DeTox for DT.

**TABLE 1:** Software used to compute the toxicity descriptors for the tacrine derivatives.

	**LD**_**50**_	M	DT	DILI
admetSAR3.0 [72]	X	X		X
ADMETlab3.0 [73]		X		X
ProTox3.0 [74]		X		X
DeTox [75]			X	
DILI‐predictor [76]				X
HTP [77]				X

The synthetic accessibility (SA) was predicted with the *Ambit‐SA* [78] software. It quantifies SA with values ranging from 0 to 100. The higher the SA values the easier it is to synthetize a molecule.

The chemical space was sampled by calculating the selection score (*S* ^*S*^) through the *CADMA‐Py* [49, 50, 66] program. *S* ^*S*^ accounts for bioavailability, toxicity, and manufacturability of a molecule. Higher values of *S* ^*S*^ correspond to compounds with better drug‐like behavior, lower toxicity, and easier synthesis. This score is obtained from three terms that includes the ADME properties, toxicity, and synthetic accessibility (Text S1).

### 2.3 Reactivity Descriptors

Full geometry optimizations and frequency analysis were carried out at the M05‐2X/6‐31+G(d) level. M05‐2X [79] is a density functional theory (DFT) approach designed for thermochemistry, kinetics, and noncovalent interactions [79]. It also has been used successfully to obtain high quality kinetic data for radical‐molecule reactions in aqueous solution [80, 81], bond‐dissociation energies [82], and p*K*_a_ values [83, 84, 85]. All the structures were identified as local minima (i.e., they exhibit no imaginary frequencies). Open‐shell systems were studied with an unrestricted approach. The solvent (water) was mimicked through the implicit solvation model based on density (SMD) [86]. All the electronic structure computations were done with *Gaussian 09* Rev D.01 [87]. The energetic analysis was performed with *Eyringpy* [88].

Ionization energies (IE) and bond dissociation energies (BDE) were computed using the adiabatic SCF scheme, and reaction enthalpies, respectively. Particularly, the for the BDEs all the most probable sites for H donation were considered (i.e., the OH and the sp^3^ carbons). This was done to investigate if the derivatives behave as antioxidants through the single electron transfer (SET) and hydrogen atom transfer (*f*‐HAT) mechanisms.

Deprotonation routes were obtained with the Marvin [89] software (available at https://playground.calculators.cxn.io/) and were confirmed using electronic structure calculations considering all the possible deprotonation sites. The p*K*_a_ values were estimated employing the fitting parameters approach. The used fitted parameters (*m* and *C*_0_) are the ones for phenols (0.313, −80.020) and amines (0.475 and −124.072) [83].

### 2.4 Molecular Docking

The structure of each used protein, co‐crystallized with its natural substrate (NS) and a reference drug (RD), was obtained from the Protein Data Bank [90]. Their data are summarized in Table 2. The AChE misplaced loop sections (256–261 and 493–496 residues) were fixed with Modeller [95]. The energy was minimized with Swiss‐PDB Viewer 4.1 [96]. The proteins were prepared by eliminating water molecules, inhibitors, and species of non‐interest with AutoDockTools4 [97]. The atomic charges of the NSs, RDs, and ligands (i.e., tacrine and its derivatives) were computed using the NBO protocol at the M05‐2X [79]/6‐31+G(d) level with *Gaussian 09* Rev D.01 [87]. Molecular docking was carried out with Autodock Vina 1.2.7 software [98, 99]. A gradient optimization algorithm was used for the active site that was centered at *x* = −16.30, *y* = −43.83, and *z* = 30.17 with a grid size of 21 × 21 × 21 Å for AChE; *x* = 166.14, *y* = 174.34, and *z* = 216.82 with a grid size of 15 × 15 × 15 Å for NMDAr; *x* = −10.49, *y* = 40.60, and *z* = 61.64 Å with a grid size of 15 × 15 × 15 Å for COMT; and *x* = 51.81, *y* = 156.34, and *z* = 28.15 with a grid size of 15 × 15 x 15 Å for MAO‐B. The docking score (i.e., the binding energy, $\Delta G_{B}$) and the Similarity Interaction Score [100] (*S*_*SI*_, Text S1) were estimated for each protein–ligand complex. The binding energy was weighted (${\Delta G}_{B}^{W}$, Text S1) with the molar fractions of the relevant acid–base species (i.e., those with molar fraction ≥1.0%) at *p*H = 7.4. The best‐docked pose of the most stable protein–ligand complex was analyzed and drawn with Discovery Studio [101]. Redocking was performed with *Chimera* 1.17.3 [102]. The RMSD values and the binding energies for the complexes AChE‐donepezil, NMDAr‐memantine, COMT‐tolcapone, and MAO‐B‐safinamide, were 2.8, 0.6, 1.6, and 1.8 Å and −12.0, −5.8, −7.6, and −10.0 kcal mol^−1^, respectively. They agree well with the observed values of IC_50_, Ki or $\Delta G_{B}$ [91, 92, 93, 94], which supports the reliability of the used docking methodology. The RMSD value obtained for AChE‐donepezil indicates moderate reproduction of the crystallographic conformation. This value could be influenced by the elongated and flexible structure of donepezil, which spans different regions of the AChE binding site. Therefore, the AChE docking results were interpreted with caution and supplemented with molecular dynamics simulations for a representative AChE–TAC complex.

**TABLE 2:** Data of the proteins used in molecular docking.

	PDB ID	Natural substrate	Reference drug
AChE	4EY7 [91]	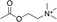 Acetylcholine	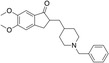 Donepezil
NMDAr	7SAD [92]	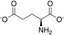 Glutamate	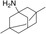 Memantine
COMT	3S68 [93]	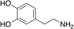 Dopamine	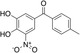 Tolcapone
MAO‐B	2V5Z [94]	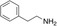 Phenylethylamine	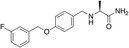 Safinamide

### 2.5 Molecular Dynamics

Molecular dynamics (MD) simulations were performed on the selected AChE:TAC‐674 and MAO‐B:TAC‐678 complexes. Starting structures were obtained from the docking best poses, and input files were prepared using CHARMM‐GUI [103]. The systems were described using the CHARMM36 force field [104] and simulated with GROMACS 2024.2 [105]. The complexes were placed in a water box using the TIP3P model [106], neutralized with counterions, and supplemented with 0.05 M of NaCl. Long‐range electrostatic interactions were treated using the Ewald method [107], while short‐range electrostatic and van der Waals interactions were calculated using a 1.2 nm cut‐off. For the MAO‐B system, the flavin adenine dinucleotide (FAD) cofactor was retained during system preparation.

Prior to production, each system was minimized using the steepest descent algorithm, followed by constrained equilibrium under NVT and NPT conditions. Temperature was controlled with the rate‐scaling thermostat [108], and pressure was maintained at 1.0 bar using a standard barostat [109]. Covalent bonds involving hydrogen atoms, or all bonds when required for the FAD‐containing (MAO‐B) system, were constrained using the LINCS algorithm [110], allowing an integration step of 2 fs. Production simulations were run for 100 ns without positional constraints.

## 3 Results and Discussion

### 3.1 Sampling the Chemical Space

By introducing —OH, —OCH_3_, —CHO, —SH, and —COOCH_3_ groups into the R_1_–R_4_ sites, 1295 tacrine derivatives (Table S2) were built. For the three combinations, the ADME properties, toxicity, and synthetic accessibility values of these derivatives were compared against a reference set of compounds that have been used as neuroprotectors or are in advanced clinical phases. The values of the ADME properties, toxicity, synthetic accessibility, and selection scores for the derivatives (including tacrine) and the reference set for combinations 1–3 are provided as Supporting Information, in Tables S3–S5 and S6, respectively.

**FIGURE 1:**
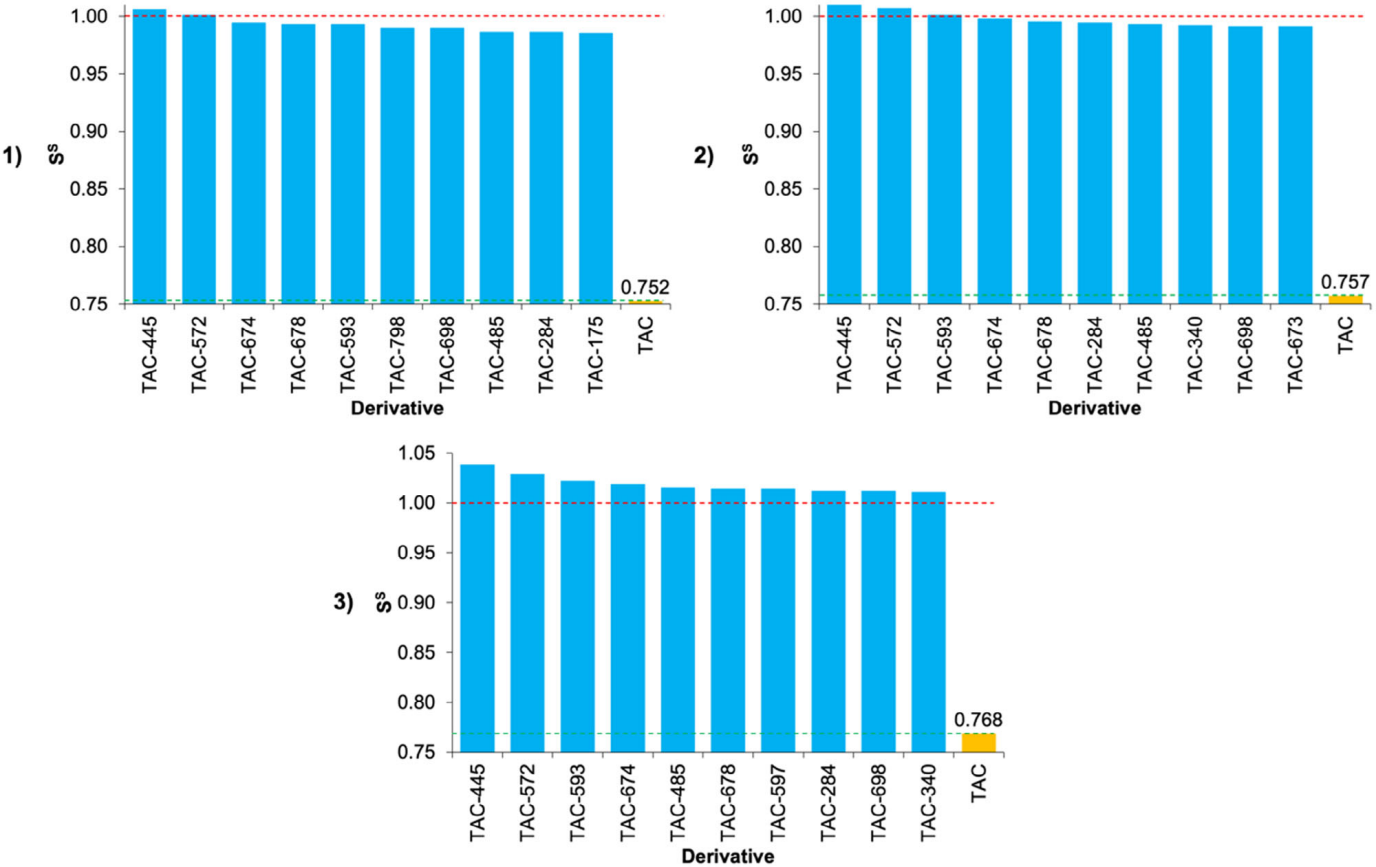
Selection scores *S* ^*S*^ of the tacrine derivatives with the best drug‐like behavior for combinations 1, 2, and 3. The green line is the value for tacrine, and red line is the average value of the reference set.

Because of the large amount of the studied molecules, we present only the *S* ^*S*^ of the first ten most promising ones (Figure 1). Two, three, and 10 derivatives (for combinations 1, 2, and 3, respectively) are better scored than both the parent molecule and the average value of reference set (*S* ^*S*^ = 1.0). Remarkably, TAC‐445, TAC‐572, TAC‐674, TAC‐678, TAC‐593, TAC‐698, TAC‐485, and TAC‐284 are predicted by the three software combinations used, among the 10 best scored ones, which shows the reliability of our results.

**FIGURE 2:**
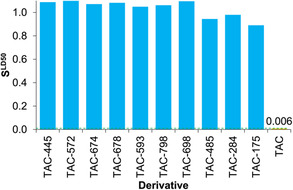
Oral rat 50% lethal dose score (*S* ^*LD50*^) of the tacrine derivatives with the best drug‐like behavior computed with admetSAR3.0. The green line is the value for tacrine.

**FIGURE 3:**
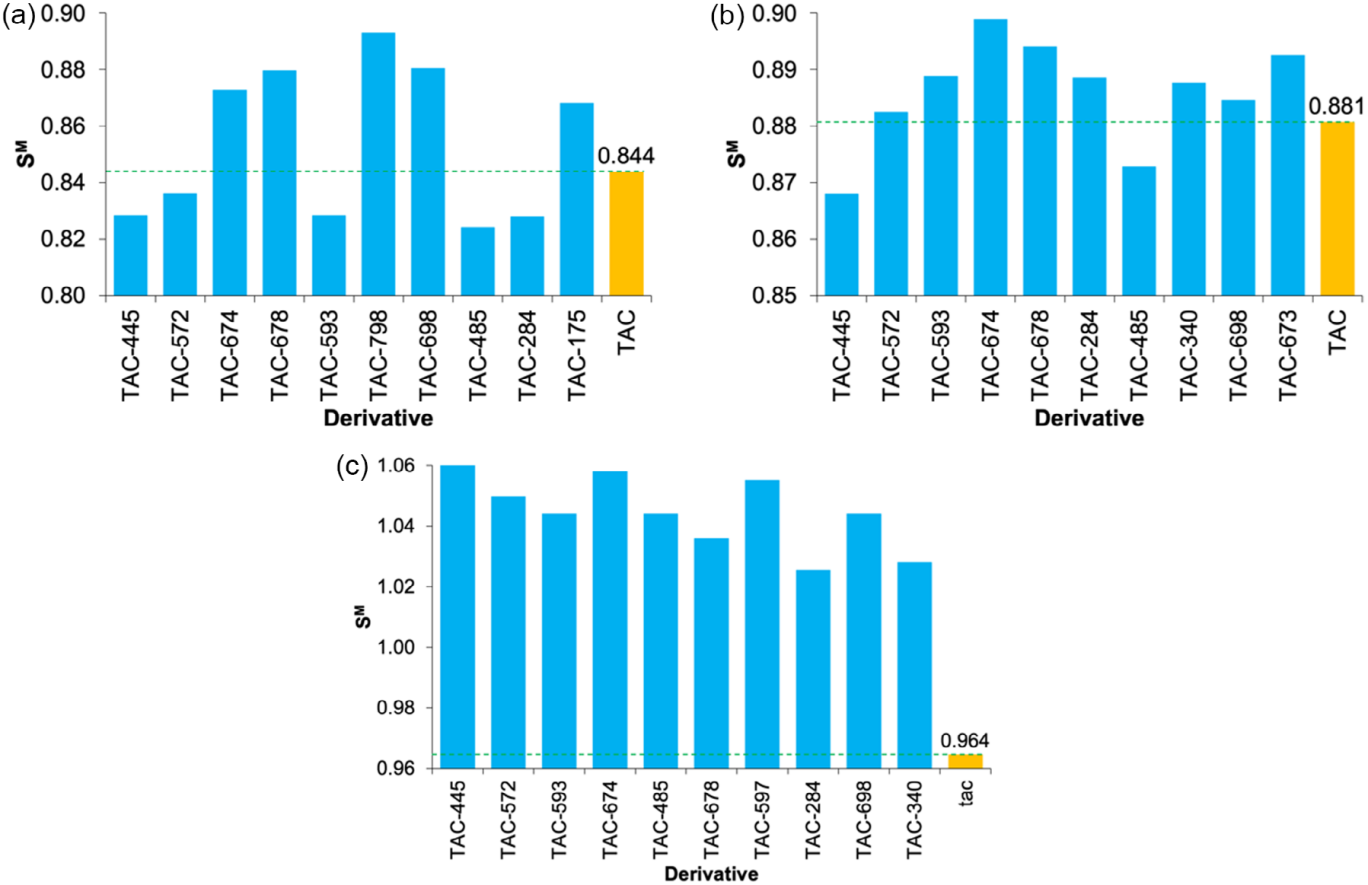
Mutagenicity score (*S* ^*M*^) of the tacrine derivatives with the best drug‐like behavior computed with (a) admetSAR.3.0, (b) ADMETlab3.0, and (c) ProTox3.0. The green line is the value for tacrine.

**FIGURE 4:**
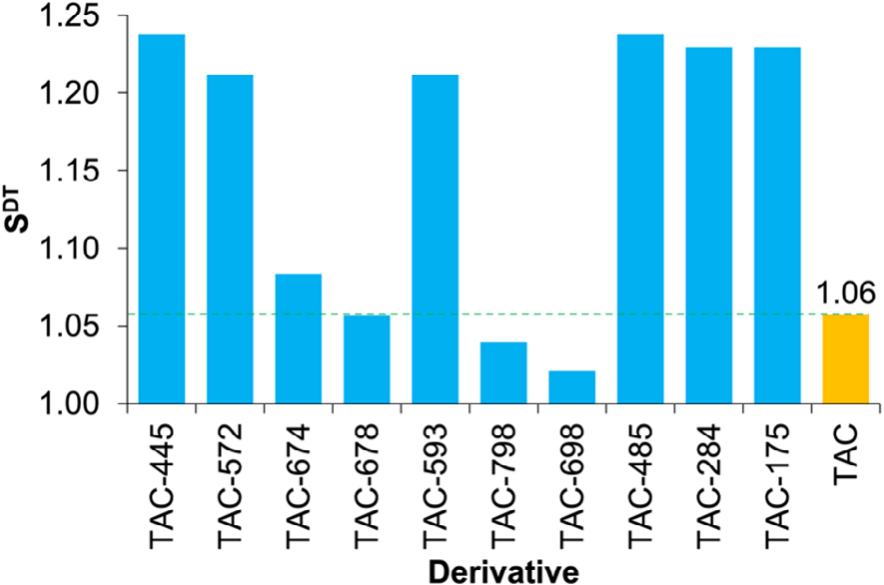
Developmental toxicity score (*S* ^*DT*^) of the tacrine derivatives with the best drug‐like behavior computed with DeTox. The green line is the value for tacrine.

The toxicity scores *S* ^*LD50*^, *S* ^*M*^, and *S* ^*DT*^ were estimated as explained in Text S1. Their values for tacrine derivatives are depicted in Figures 2–4, respectively. TAC‐445, TAC‐572, TAC‐674, TAC‐678, TAC‐593, and TAC‐698 exhibit higher *S* ^*LD50*^ values when compared to the reference set (*S* ^*LD50*^ = 1.0) and tacrine (*S* ^*LD50*^ = 0.006) (Figure 2). Hence, they are significantly less toxic to rats than the parent molecule. In the case of *S* ^*M*^ higher values correspond to less mutagenic compounds. In the three combinations only TAC‐674, TAC‐678, and TAC‐698 repeatedly appear as less mutagenic than tacrine (Figure 3). Finally, for *S* ^*DT*^ higher values correspond to compounds less toxic for development. The ten derivatives have a developmental toxicity lower than the reference set (see Figure 4). Besides, only TAC‐698 and TAC‐798 have worse developmental toxicity with respect to tacrine.

As previously mentioned, tacrine was banned from the market because of its hepatotoxicity. Thus, we investigated this descriptor by calculating the DILI of the first ten tacrine derivatives (Table S7). ProTox3.0 predicts all these derivatives and tacrine as non‐hepatotoxic, while DILI‐Predictor and HTP classify them all as hepatotoxic. By ADMETlab3.0 and admetSAR.3.0 some derivatives are negative. Therefore, it is impossible to reliably establish if the tacrine derivatives are potentially hepatotoxic using the available software. This particular risk needs to be experimentally evaluated after the most promising candidates are synthesized.

**SCHEME 2:**
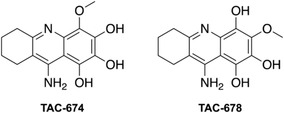
Two‐dimensional structures of the best‐scored tacrine derivatives.

Based on the above discussed toxicity results, TAC‐674 and TAC‐678 (Scheme 2) are predicted to have the best desirable features of an oral drug (i.e., bioavailability, adequate permeation, low toxicity, and easy manufacturability). Hence, they were chosen for the next stage of the investigation, i.e., the evaluation of their potential as antioxidants.

### 3.2 Acid‐Base Equilibria and Antioxidant Activity

Unveiling the acid–base equilibria is essential to determine if a molecule can cross a biological barrier through passive diffusion. The p*K*_a_ values and deprotonation routes of the two selected tacrine derivatives are given in Figure S1. Molar fractions in percentage, at blood pH, are listed in Table 3. The distribution diagrams are depicted in Figure S2.

**TABLE 3:** Estimated p*K*_a_ and molar fractions (%), M*f*_(*q*)_, at pH = 7.4. The *q* in the acronym corresponds to the charge of the acid–base species.

Derivative	**p*K*_a_**_**1**_	**p*K*_a_**_**2**_	**p*K*_a_**_**3**_	**M*f***_**(+1)**_	**M*f***_**(0)**_	**M*f***_**(−1)**_	**M*f***_**(−2)**_
TAC	9.9			99.7	0.3		
TAC‐678	7.1	8.8	12.0	30.0	67.0	3.0	<0.1
TAC‐674	5.1	10.3	11.6	0.5	99.4	0.1	<0.1

The neutral species of the selected derivatives are highly abundant at physiological pH. Consequently, it is expected that the chosen compounds can easily cross the biological barriers by passive diffusion. However, the cationic forms of TAC‐678 have significant molar fractions, and its anionic forms are present at non‐negligible molar fraction (≥1.0%). Therefore, these anionic species might enhance antioxidant activity.

**FIGURE 5:**
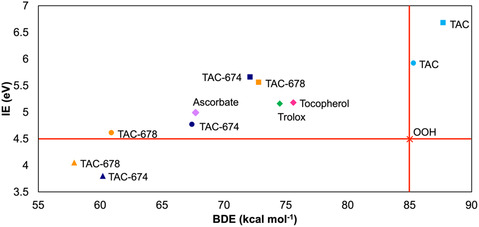
The electron and hydrogen donating ability map (eH‐DAMA) of the most promising derivatives and their most abundant acid–base species at physiological pH. Cationic, neutral, and anionic forms are depicted by squares, circles, and triangle figures, respectively.

IEs and BDEs were computed for the acid–base species with non‐negligible molar fractions (i.e., ≥1.0%) (Table S8). IE and BDE account for the capability of a molecule to donate one electron and one H‐atom, respectively. Therefore, they were employed to assess the efficacy of tacrine derivatives as free radical scavengers through SET and *f*‐HAT mechanisms, respectively. The electron and hydrogen donating ability map for antioxidants (eH‐DAMA, Figure 5) was built using the IEs and BDEs. Hence, this graph serves to predict the probability of a molecule to act simultaneously as an electron or H donor by the SET or *f*‐HAT mechanisms, respectively. Trolox, ascorbic acid, and tocopherol were used as reference antioxidants. H_2_O_2_/^•^OOH pair is the oxidant target. The best radical scavengers exhibit lower IE and lower BDE than the oxidant target [111, 112].

According to the eH‐DAMA, the anionic forms of TAC‐674 and TAC‐678 are the most efficient for deactivating hydroperoxyl radicals *via* SET and *f*‐HAT mechanisms. They are also expected to exhibit a better performance than the parent molecule, trolox, tocopherol, and ascorbic acid.

### 3.3 Multi‐Target Activity

The potential interaction of tacrine derivatives with neuroprotection‐related targets was investigated by molecular docking simulations. The interaction of tacrine derivatives with key proteins (i.e., AChE, NMDAr, COMT, and MAO‐B) was assessed by computing the weighted binding energies (${\Delta G}_{B}^{W}$) and multi‐target scores ($S^{MT}$) (Text S1). These parameters were compared with those of the natural substrates (i.e., endogenous ligands or physiological substrates) of such proteins and some reference drugs. The ${\Delta G}_{B}^{W}$ values of the best derivatives, NSs, and RDs are provided in Table 4. The complete docking results are listed in Table S9. Because docking scores are target‐dependent, comparisons should be understood only within each protein system against the corresponding NS and RD, rather than as direct quantitative comparisons between different proteins.

**TABLE 4:** Weighted binding energies (in kcal mol^−1^) for TAC, the selected derivatives, natural substrates, and reference drugs.^a^

	${\Delta G}_{B}^{W}$			
Compound	**AChE**	**NMDAr**	**COMT**	**MAO‐B**
TAC	−8.7	−6.6	−5.5	−9.3
TAC‐678	−8.7	−7.0	−5.7	−8.9
TAC‐674	−9.7	−7.0	−6.1	−8.6
NS	−4.9	−5.0	−5.4	−6.0
RD	−12.0	−5.8	−7.6	−10.0

a Protein (NS, RD): AChE (acetylcholine, donepezil), NMDAr (glutamate, memantine), COMT (dopamine, tolcapone), and MAO‐B (phenylethylamine, safinamide).

The ${\Delta G}_{B}^{W}$ values of the tacrine derivatives are more negative than those of NSs for all the analyzed proteins, which suggests a potential multitarget interaction profile relevant for neuroprotective activity. For AChE, the selected compounds have ${\Delta G}_{B}^{W}$ values significantly more negative than ACh, but a moderate performance relative to the clinical standard donepezil. TAC‐674 stands out for its predicted binding profiles. It is noteworthy that the predicted binding energy for the AChE–tacrine complex (−8.7 kcal mol^−1^) agrees closely with experiment (−8.6 kcal mol^−1^) [113], supporting the reliability of our computational protocol. As for the NMDA receptor, the derivatives have more favorable docking scores than those of glutamate and memantine, indicating possible interaction with the ion channel binding site. In the case of COMT, the binding energies of the derivatives are similar to dopamine, but lower than tolcapone, suggesting potential interaction with this enzyme. For MAO‐B, the tacrine derivatives exhibit more favorable affinities than phenylethylamine and values close to that of safinamide, supporting their prioritization as promising candidates. Overall, within each specific protein comparison, tacrine derivatives showed favorable predicted binding relative to the corresponding physiological substrates and, in some cases, binding scores close to those of the respective reference drugs. TAC‐678 and TAC‐674 stand out as balanced candidates according to these protein‐specific docking comparisons, particularly for AChE and NMDAr.

**FIGURE 6:**
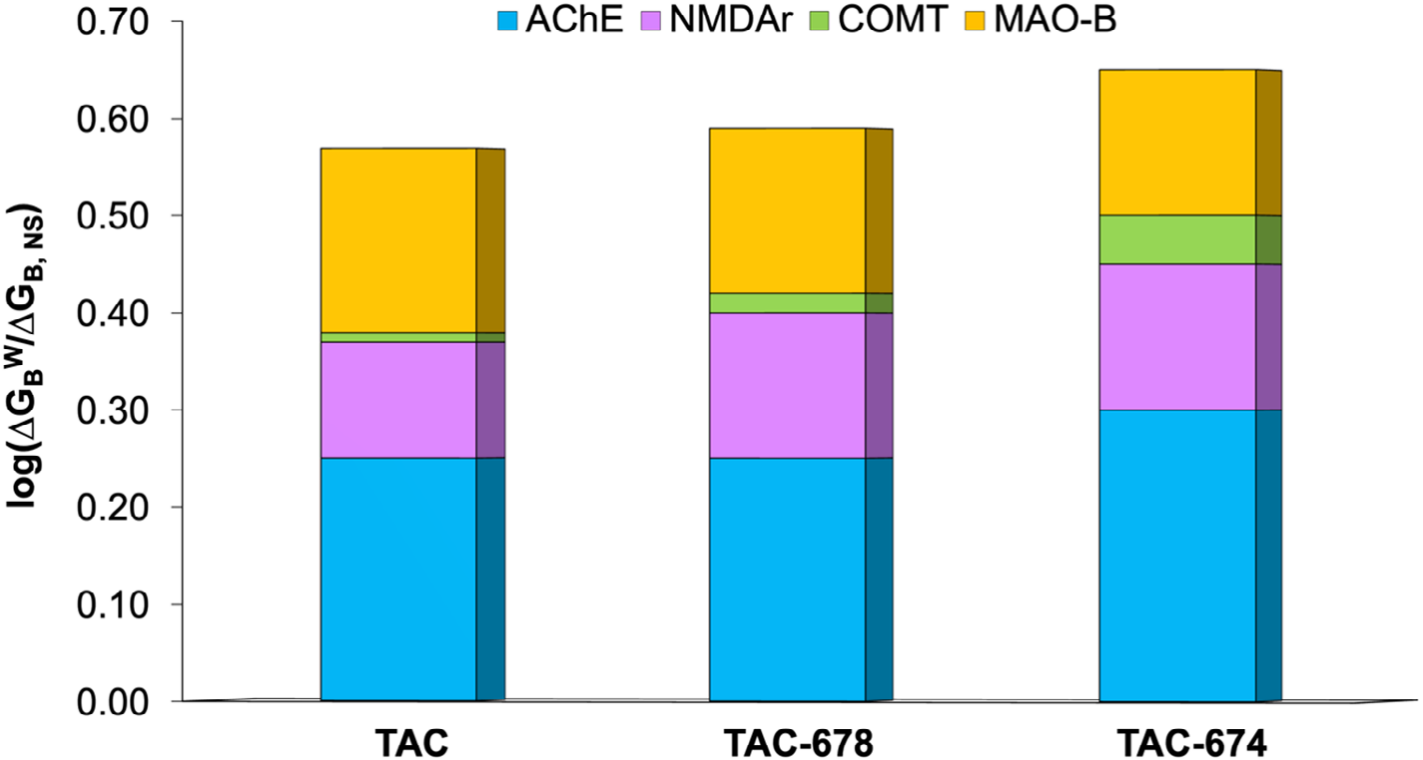
Multitarget efficiency plot of tacrine and its derivatives, relative to the natural substrates.

A multitarget efficiency plot (Figure 6) was constructed to provide a visual assessment of the polypharmacological profile of the designed compounds, based on their binding efficiency compared with the corresponding NSs. This plot and the $S^{MT}$ should be used only as compound‐prioritization tools within the docking workflow and should not be interpreted as evidence of competitive inhibition. Higher values correspond to more favorable docking performance relative to the corresponding NS. The analyzed derivatives showed more favorable predicted binding performance than tacrine toward AChE, NMDAr, and MAO‐B.

All the selected derivatives interact more strongly with the proteins than the endogenous ligands, particularly with AChE, NMDAr, and MAO‐B, and to a lesser extent for COMT. Thus, this graph supports that these TAC derivatives show a predicted polygenic profile. It also indicates that TAC‐674 is the most promising candidate according to this descriptor. On the other hand, the plot relative to RDs is depicted in Figure S3. Tacrine and its derivatives show more favorable docking scores only for the NMDA receptor with respect to the reference drug. In this regard, TAC‐678 shows the highest predicted score for this receptor compared with memantine, which is used to treat AD [92].

In addition to protein‐ligand docking and multitarget scores, it is also useful to consider the amino acid residues involved in the protein–ligand interaction as well as their nature because they contribute to the stability of the complex and provide structural insights into binding modes. To this end, we calculated the similarity interaction score [100] ($S_{SI}$, Text S1), which allows comparison of the pattern of interactions of a candidate compound with respect to a reference ligand (i.e., NS or RD). $S_{SI}$ quantifies the overlap between residues involved in the interactions as well as the type of interaction (e.g., hydrogen bonds, π interactions, among others). It is expressed as a normalized value between 0 (no similarity) and 1 (complete match). Conceptually, $S_{SI}$ is related to established protein–ligand interaction fingerprinting methods, such as PLIF, IFP, and SIFt, as it compares ligand‐binding conformations using residue–level interaction patterns [114, 115, 116]. In this work, $S_{SI}$ condenses residue matching and interaction type agreement with a selected reference ligand into a single similarity value. Its main practical advantage in the present analysis lies in its simplicity and transparency since individual matching terms can be directly traced. It is important to note that $S_{SI}$ is not used here as an independent predictor of bioactivity, binding strength, or enzyme inhibition. However, to establish its predictive capacity, formal validation of *S*_*SI*_ would be necessary by comparing it with experimental bonding data and/or crystallographic interaction fingerprints. Such validation is beyond the scope of this study. Instead, it is used as a descriptor complementary to the docking score to qualitatively prioritize compounds that exhibit interaction patterns compatible with the relevant regions of the protein binding site. For comparative purposes, an internal reference value $\left(S_{SI}^{Ref}\right)$ was estimated from the average *S*_*SI*_ calculated between the natural substrates and the corresponding reference drugs. This value was used as an internal qualitative guide to contextualize the similarity of interaction patterns within this dataset. In this case, the value of $S_{SI}^{Ref}$ is 0.38. In practice, compounds with values close to or above the $S_{SI}^{Ref}$ are interpreted as showing partial interaction‐pattern similarity to the reference ligand within this dataset. This outcome reflects that the index favors poses that are functionally coherent within the binding site, rather than simply having high docking energies. Table 5 summarizes the $S_{SI}$ values. The stabilizing interactions between tacrine derivatives and the analyzed proteins and the parameters used in the calculation of the score are provided in Tables S10 and S11, respectively.

**TABLE 5:** Similarity interaction score ($S_{SI}$) for the selected tacrine derivatives.^a^

	AChE		NMDAr		COMT		MAO‐B	
Compound	NS	RD	NS	RD	NS	RD	NS	RD
TAC	0.14	0.25	N.D.	0.63	0.42	0.36	0.38	0.28
TAC‐678	0.43	0.50	N.D.	0.88	0.00	0.14	0.38	0.50
TAC‐674	0.36	0.50	N.D.	0.50	0.50	0.57	0.50	0.28

Abbreviation: N.D., not determined.

a $S_{SI}^{Ref}=0.38$ (Text S1).

In general, the highest $S_{SI}$ values of the tacrine derivatives were found when compared with the RDs. Nevertheless, all the chosen derivatives show favorable $S_{SI}$ values for NMDAr. TAC‐674 and TAC‐678 show more promising interaction profiles, compared to tacrine, with values above the $S_{SI}^{Ref}$ for several key targets. TAC‐674 stands out for its compatibility with the endogenous ligands (acetylcholine, dopamine, and phenylethylamine) and RDs (i.e., donepezil, tolcapone, memantine, and safinamide), reinforcing its multitarget potential. TAC‐678 also exhibits interaction profiles similar to those of NS and RD on several targets, particularly for AChE and MAO‐B, supporting its selection as a candidate multi‐target compound. In contrast, TAC showed a more restricted profile, with lower or target‐dependent similarity scores. For the NMDA receptor, *S*_*SI*_ was calculated exclusively against memantine, as the aim of this study is to assess channel‐blocking behavior. Comparison with the endogenous agonist glutamate is therefore not pharmacologically relevant for the purposes of this study.

The interactions that stabilize the complexes were analyzed to explore the structural basis of their binding profiles. TAC‐674 and TAC‐678 derivatives combine favorable docking scores and interaction indices with promising antioxidant behavior and were therefore selected as representative candidates for further analysis.

**FIGURE 7:**
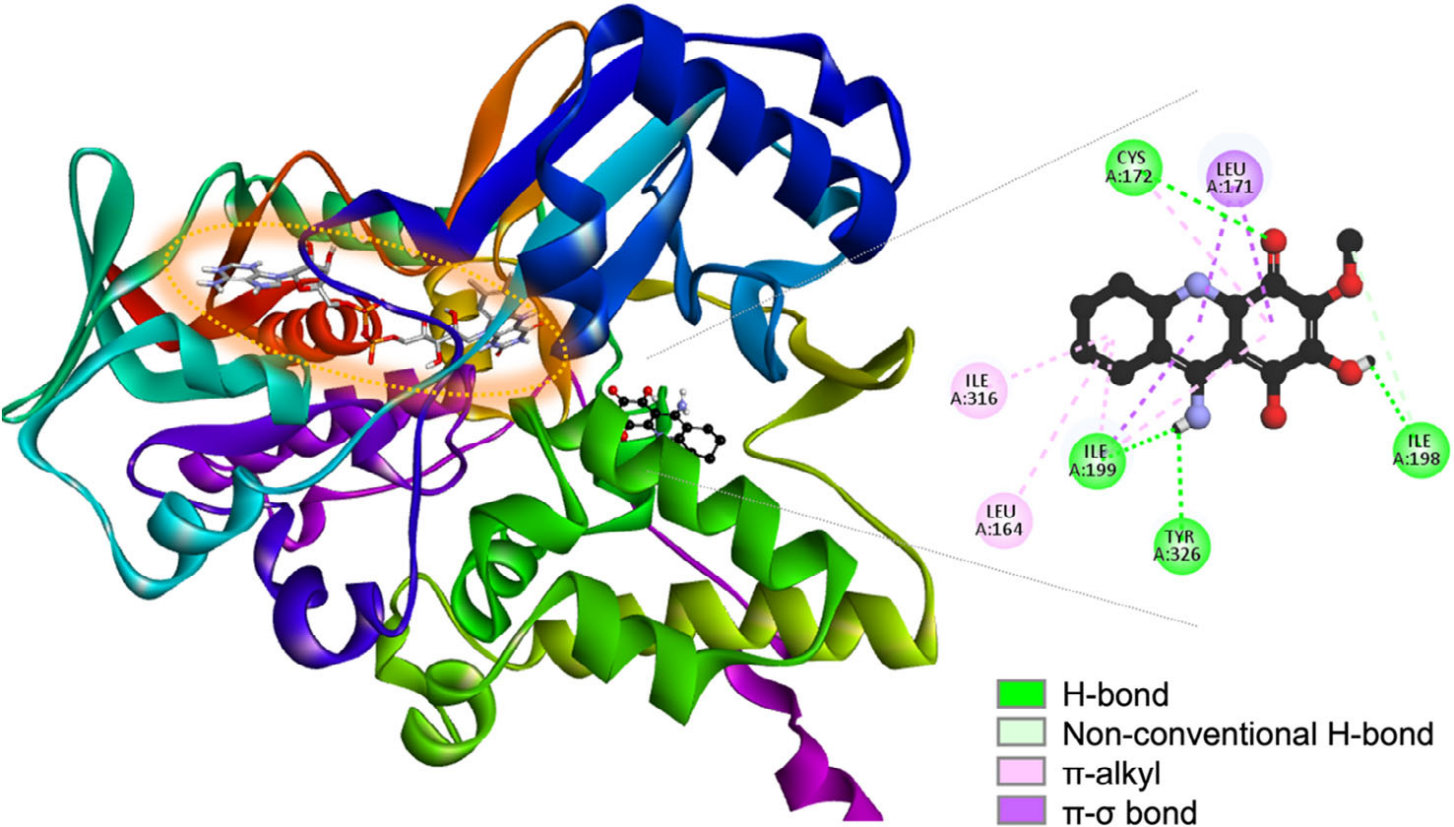
3D and 2D interaction network in the [MAO‐B:TAC‐678] complex.

Figure 7 shows the interaction profile of the [MAO‐B:TAC‐678] complex. TAC‐678 forms several contacts in the MAO‐B active site by interacting with residues involved in substrate‐specific recognition and ligand anchoring in the catalytic environment. Hydrogen bond formation was observed with Tyr326, Ile198, Ile199, and Cys172. Tyr326 is known to be critically important as part of the aromatic arc that guides entry into the MAO‐B active site [94, 113]. Additionally, hydrophobic interactions with Leu164, Ile316, Leu171, Ile199, and Cys172 as well as additional π‐sigma interactions with Leu171 and Ile199 were identified, suggesting favorable anchoring of the aromatic group of TAC‐678 in the hydrophobic cavity. These interactions may contribute to ligand retention near the FAD access region (highlighted orange region), which could limit FAD availability for endogenous amine oxidation [117, 118].

MAO‐B is responsible for the catalytic degradation of neuroactive amines such as phenylethylamine and, in part, dopamine. Its inhibition is associated with an increase in the concentration of these neurotransmitters in the neuronal network, which generally results in neuroprotective, antidepressant, and antiparkinsonian effects [119]. Inhibition of this enzyme has also been proposed as a strategy to reduce oxidative stress‐related neurodegeneration [120]. The $S_{SI}$ value of TAC‐678 with respect to the reference ligand is above the study‐specific internal reference value, suggesting partial similarity with the binding pattern of safinamide and supporting its prioritization for further evaluation against MAO‐B.

**FIGURE 8:**
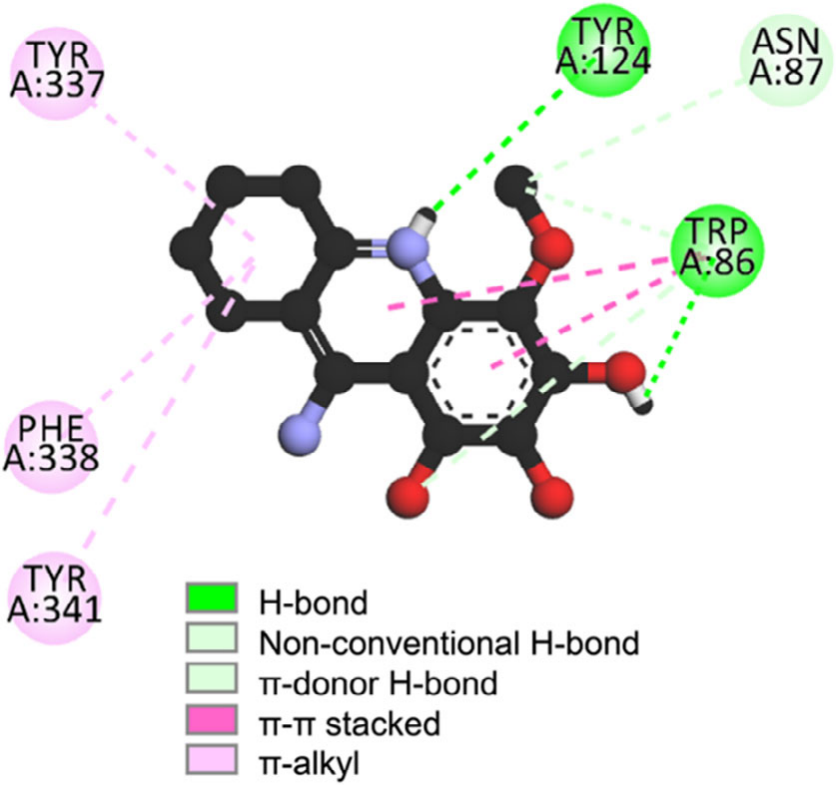
3D and 2D interaction map in the [AChE:TAC‐674] complex.

The interactions that stabilize the complex [AChE:TAC‐674] are depicted in Figure 8. TAC‐674 exhibits a binding mode that combines specific interactions with key residues involved in AChE function. H‐bonds were observed with Trp86, Asn87, and Tyr124; these amino acids are close to or belong to the active site. In addition, various π‐alkyl interactions and π‐π stacking were found with Tyr337, Phe338, and Tyr341, associated with the peripheral anionic site. This region is important in allosteric modulation and ACh scavenging [53]. This binding mode suggests that this derivative can interact with both catalytic and peripheral region of AChE. Similar interaction patterns have been reported for several multifunctional AChE inhibitors [55, 91, 121].

The $S_{SI}$ value and the topology of interactions suggest that TAC‐674 could act as a dual‐site interacting compound, potentially affecting catalysis and allosteric regulation. However, this proposed dual‐site interaction behavior is a docking‐based hypothesis that requires experimental validation. Such a mechanism would be relevant for potential AD‐oriented compounds, where AChE inhibition is sought to enhance cholinergic signaling [122]. Furthermore, peripheral site occupation has been related to a reduction in the formation of A*β* plaques [123].

**FIGURE 9:**
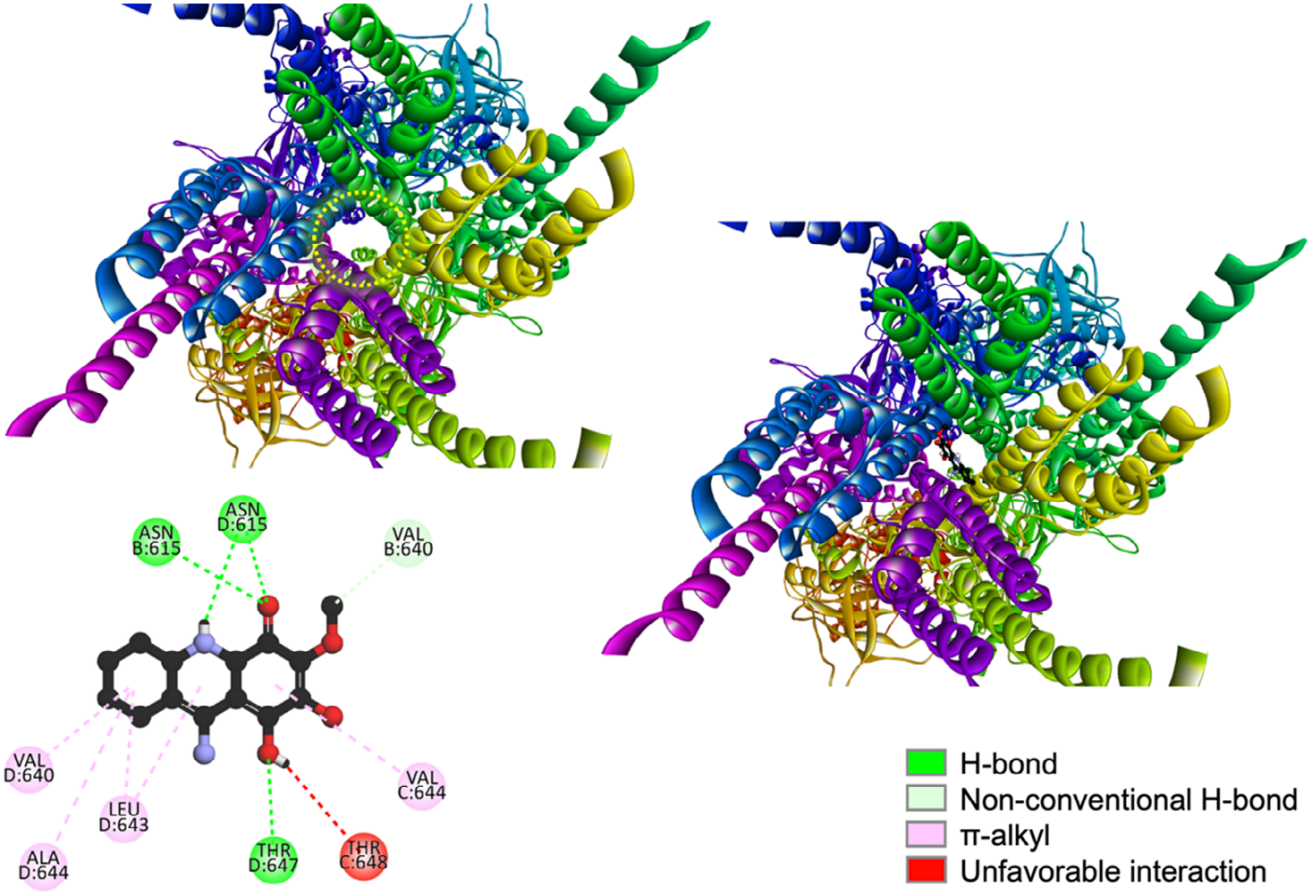
3D and 2D interaction network in the [NMDAr:TAC678] complex.

Figure 9 presents the interaction network formed by the [NMDAr:TAC‐678] complex. TAC‐678 binds to the NMDA receptor in a region close to the ion channel (yellow circle), with an interaction profile that suggests a possible overlap with the binding region of known channel blockers such as memantine or ifenprodil [99, 108]. Ligand binding occurs through several hydrogen bonds with the polar residues Asn(B):615, Asn(D):615, and Thr(D):647. Additionally, several unconventional hydrogen bonds with Val(B):640 contribute to anchoring. Finally, several hydrophobic interactions with Val(D):640, Ala(D):644, and Leu(D):643 reinforce this conformation. Thr(C):648 represents a steric hindrance, which could interfere with the stability of the complex. The arrangement of the ligand and the variety of chains involved (B, C, D) suggest that TAC‐678 is positioned between the different subunits of the NMDAr complex, in a region close to the ion channel. This binding pattern resembles that of memantine, a known voltage‐gated channel blocker that prevents Ca^2+^ influx without directly competing with ligands at the orthosteric site [109]. It is important to emphasize that the NMDAr is a multisubunit transmembrane ion channel, and therefore, the docking results for this target should be interpreted with caution, as the lipid environment, ion channel dynamics, receptor flexibility, and membrane‐associated conformational effects are not captured in the rigid receptor docking model used in this work.

The $S_{SI}$ value for TAC‐678 suggests that this derivative share certain interaction characteristics with memantine, supporting its prioritization as a candidate for functional validation against NMDAr. This is particularly important because excessive or nonselective NMDAr blockade can be pharmacologically undesirable and associated with adverse effects. In this regard, the observed steric hindrance could guide structural optimization, because it may affect conformational stability, but it could also be relevant for preventing excessive or persistent NMDAr binding, given that a stronger interaction with the channel is not necessarily pharmacologically advantageous.

**FIGURE 10:**
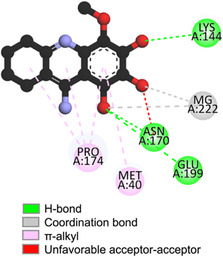
2D interaction map in the [COMT:TAC‐674] adduct.

Analysis of the binding mode of TAC‐674 with the COMT enzyme, presented in Figure 10, shows that this derivative is located within the catalytic site. A coordination bond is established with the Mg^2+^ ion, an essential cofactor for catechol methylation. This interaction, along with hydrogen bonds with Lys144, Asn170, and Glu199, generates a binding pattern that resembles that observed with inhibitors such as tolcapone and entacapone. This conformation suggests a competitive interaction that could be compatible with potential inhibitory mechanism hypothesis [107, 124]. Steric hindrance is also observed with Asn170, which could affect the dynamics of substrate entry.

Further stabilization of the complex is favored by hydrophobic interactions with Pro174 and Met40, which may contribute to ligand binding in the catalytic cavity. These observations are consistent with the literature on COMT and its inhibitors, where the joint participation of metal‐donor bonds, hydrogen bonds, and hydrophobic interactions are key for effective inhibition [125]. Again, the *S*_*SI*_ values of TAC‐674 for COMT indicate an interaction pattern that resembles the patterns observed for NS (dopamine) and RD (tolcapone) with scores above the internal reference value, $S_{SI}^{Ref}$. These results support the computational prioritization of TAC‐674 as a putative modulator of catecholamine metabolism in the central nervous system.

The predicted profiles of the tacrine analogs suggest an interaction profile consistent with neuroprotection‐related processes and involving multiple targets related to neurodegeneration. From a mechanistic perspective, their potential interaction with AChE could be associated with procognitive effects by preserving synaptic ACh levels. Likewise, their interaction with MAO‐B and COMT could be related to the regulation of catecholaminergic neurotransmitters, including dopamine metabolism. Finally, their possible interaction in the NMDAr channel region suggests a modulatory role in glutamate excitotoxicity, relevant to AD and PD. Therefore, the selected tacrine derivatives exhibit a computationally predicted multifunctional profile that supports their selection as candidates for further development and experimental evaluation. Nevertheless, further studies are required to confirm or refute these proposed mechanisms and their pharmacological relevance. To complement the analyses based on molecular docking and to examine the dynamic stability of representative binding poses, MD simulations were performed for representative protein‐ligand complexes.

### 3.4 Molecular Dynamics of [MAO‐B:TAC‐678] and [AChE:TAC‐674] Complexes

**FIGURE 11:**
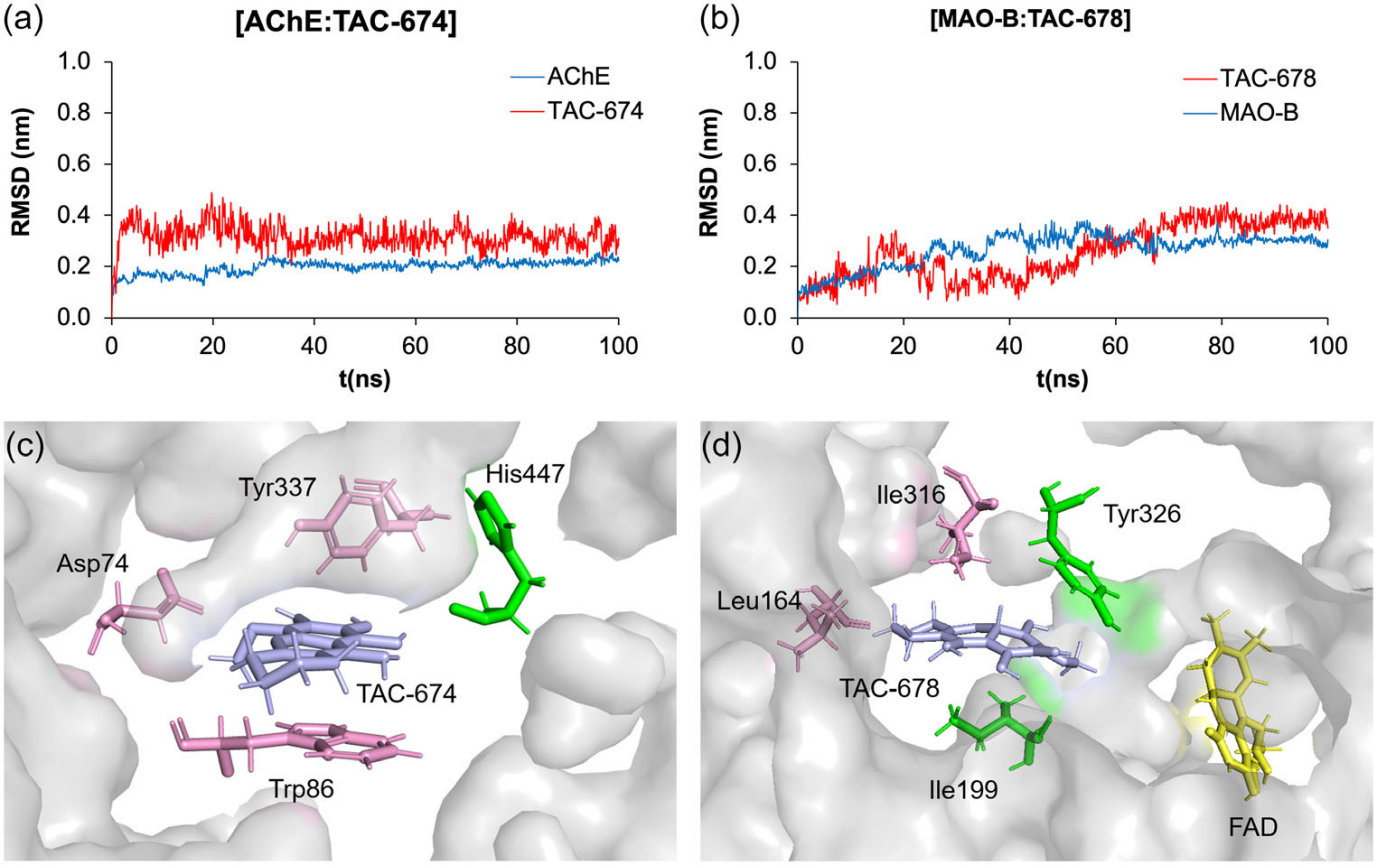
RMSD and representative pose protein ligand complexes MD. (a) RMSD value of AChE and TAC‐674 fit to protein. (b) RMSD value of MAO‐B and TAC‐674 fit to corresponding protein. (c) Representative pose of the cluster (41.0 ns) in the MD of the [AChE:TAC‐674] complex. (d) Representative pose of the cluster (88.9 ns) in the MD of the [MAO‐B:TAC‐678] complex.

To assess the stability of the docking conformations, 100 ns molecular dynamics simulations were performed for the [AChE:TAC‐674] and [MAO‐B:TAC‐678] complexes. These systems were selected because they exhibit antioxidant behavior, advantageous docking scores, and favorable interaction profiles. Additionally, AChE and MAO‐B represent enzyme targets with significance for the desired multitarget neuroprotective profile. Table 6 presents a quantitative summary of the parameters studied in the MD simulation, and Figure 11 presents some relevant plots for discussing the stability and interaction patterns of these complexes.

**TABLE 6:** Summary average parameters from the 100 ns MD simulations.

	Complex
Values	[MAO‐B:TAC‐678]	[AChE:TAC‐674]
Protein RMSD (nm)	0.28 ± 0.01	0.20 ± 0.03
TAC RMSD (nm)	0.23 ± 0.09	0.32 ± 0.05
Protein RMSF (nm)	0.18 ± 0.09	0.11 ± 0.07
TAC‐pocket distance (nm)	0.12 ± 0.06	0.53 ± 0.04
Occupancy (>90%)	Leu171, Gln206, Ile199, Tyr326, Phe168, Leu164, Trp119, Ile316, Pro104, Leu167	Gly126, Trp86, Ser125, Tyr133, Leu130, Gly121, Gly448, Tyr449, Tyr337, Asp74, His447
H‐bonds	2 ± 1	<1
Contacts	160 ± 19	113 ± 14

The RMSD values for the protein (blue lines in Figure 11a,b) indicate that both enzymes remained stable during the simulations. For [AChE:TAC‐674], the RMSD remained close to 0.20 nm throughout the trajectory, suggesting that the binding of TAC‐674 did not induce relevant global structural perturbations in AChE. In the [MAO‐B:TAC‐678] system, the RMSD indicates that MAO‐B reached an equilibrated regime, particularly toward the final part of the simulation, with values around 0.30 nm. The RMSF profiles (Figures S4 and S5) support this interpretation. As expected, the largest fluctuations occurred mainly in the flexible or solvent‐exposed regions, while the binding‐site regions remained comparatively stable.

The ligand RMSD analysis (red lines in Figure 11a,b) and ligand‐pocket distance (Figures S4 and S5) shows that both TAC‐674 and TAC‐678 underwent moderate reorganization from their docked state, which is expected after MD relaxation. In [AChE:TAC‐674], the ligand remained within the binding pocket (average RMSD of 0.32 nm), but the distance to the pocket remained stable at 0.53 nm, indicating that the ligand did not leave the AChE binding region. Similar behavior was observed for [MAO‐B:TAC‐678], where the derivative exhibited a moderate increase in RMSD (0.23 nm) but remained associated with the FAD‐access channel in MAO‐B, with a ligand‐pocket distance close to 0.20 nm during the final part of the trajectory. These changes in the RMSD should be interpreted as a relaxation of the local conformation.

The analysis of contact persistence (Figures S4 and S5) provides information on the structural persistence of the binding modes. In the [AChE:TAC‐674] complex (Figure 11c), the tacrine derivative maintained contacts with residues associated with ligand recognition, including Trp86, Tyr337, Asp74, and His447. Trp86 is part of the anionic site and participates in the stabilization of aromatic inhibitors such as tacrine [126], while Tyr337 contributes to substrate orientation within the active site [126]. The contacts with Asp74 and His447 are relevant because both are in regions involved in substrate guidance and catalytic function, respectively [126]. This binding mode may partially restrict substrate access and the proper organization of the catalytic site. In the [MAO‐B:TAC‐678] complex, TAC‐678 contacted Tyr326, Ile316, Leu164, and Ile199, all residues located along the substrate entry cavity leading to the FAD cofactor [126]. This positioning suggests that TAC‐678 could obstruct or restrict substrate access to the flavin catalytic region, potentially affecting substrate oxidation.

Overall, MD simulations support the dynamic stability of the docking conformations and support the selection of TAC‐674 and TAC‐678 as multifunctional candidates. In both systems, the ligands remained associated with their respective binding regions, maintained contacts with relevant amino acids, and showed no evidence of dissociation during MD. These results provide further support for the corresponding docking findings and suggest hypotheses regarding how these tacrine derivatives could affect AChE and MAO‐B activity. However, the proposed binding modes and functional effects should be considered testable hypotheses requiring biochemical validation.

## 4 Conclusions

In this work, 1295 tacrine derivatives were designed using the CADMA‐Chem methodology. This set was sampled with a selection score (*S* ^*S*^) based on ADME properties, toxicity, and manufacturability parameters. As a result, two derivatives with the best drug‐like behavior, toxicity, and synthetic accessibility (TAC‐678 and TAC‐674) were selected as the most promising candidates. For this subset, the eH‐DAMA map was built to assess their antioxidant activity via SET and *f*‐HAT mechanisms. According to this, the anionic forms of TAC‐674 and TAC‐678 are the most efficacious free radical scavengers through SET and *f*‐HAT mechanisms. The combined computational evidence allowed TAC‐674 and TAC‐678 to be prioritized as candidates with interaction profiles compatible with binding to AChE/COMT and MAO‐B/NMDAr, respectively. TAC‐674 showed an in silico interaction profile compatible with COMT binding, highlighting an underexplored direction for tacrine‐derived compounds. Finally, MD simulations supported the dynamic stability of [AChE:TAC‐674] and [MAO‐B:TAC‐678] complexes, suggesting persistent binding without ligand dissociation. Thus, they are proposed for further investigations (theoretical and experimental).

## Funding

This study was supported by the SECIHTI (CBF2023‐2024‐1141).

## Conflicts of Interest

The authors declare no conflicts of interest.

## Supporting information

Supplementary Material

## Data Availability

The data that support the findings of this study are available on request from the corresponding author. The data are not publicly available due to privacy or ethical restrictions.

## References

[1] R. Cacabelos , “Special Issue: “New Trends in Alzheimer's Disease Research: From Molecular Mechanisms to Therapeutics: 2nd Edition,” International Journal of Molecular Sciences 26 (2025): 7175–7186. 40806308 10.3390/ijms26157175 PMC12346564

[2] Y. Luo , L. Qiao , M. Li , X. Wen , W. Zhang , and X. Li , “Global, Regional, National Epidemiology and Trends of Parkinson's Disease from 1990 to 2021: Findings from the Global Burden of Disease Study 2021,” Frontiers in Aging Neuroscience 16 (2025): 1–12. 10.3389/fnagi.2024.1498756 PMC11757241 39868382

[3] J. G. Thorp , B. L. Mitchell , Z. F. Gerring , et al., “Genetic Evidence that the Causal Association of Educational Attainment With Reduced Risk of Alzheimer's Disease Is Driven by Intelligence,” Neurobiology of Aging 119 (2022): 127–135. 35989212 10.1016/j.neurobiolaging.2022.07.011

[4] D. Su , Y. Cui , C. He , et al., “Projections for Prevalence of Parkinson's Disease and Its Driving Factors in 195 Countries and Territories to 2050: Modelling Study of Global Burden of Disease Study 2021,” British Medical Journal 388 (2025): e080952. 40044233 10.1136/bmj-2024-080952 PMC11881235

[5] V. García‐Morales , A. González‐Acedo , L. Melguizo‐Rodríguez , et al., “Current Understanding of the Physiopathology, Diagnosis and Therapeutic Approach to Alzheimer's Disease,” Biomedicines 9 (2021): 1910. 34944723 10.3390/biomedicines9121910 PMC8698840

[6] J. Zhang , Y. Zhang , J. Wang , Y. Xia , J. Zhang , and L. Chen , “Recent Advances in Alzheimer's Disease: Mechanisms, Clinical Trials and New Drug Development Strategies,” Signal Transduction and Targeted Therapy 9 (2024): 211. 39174535 10.1038/s41392-024-01911-3 PMC11344989

[7] I. Vecchio , L. Sorrentino , A. Paoletti , R. Marra , and M. Arbitrio , “The State of the Art on Acetylcholinesterase Inhibitors in the Treatment of Alzheimer's Disease,” Journal of Central Nervous System Disease 13 (2021): 11795735211029113. 34285627 10.1177/11795735211029113 PMC8267037

[8] J. He and K. Y. Tam , “Dual‐Target Inhibitors of Cholinesterase and GSK‐3β to Modulate Alzheimer's Disease,” Drug Discovery Today 29 (2024): 103914. 38340951 10.1016/j.drudis.2024.103914

[9] M. S. Uddin , A. Al Mamun , M. T. Kabir , G. M. Ashraf , M. N. Bin‐Jumah , and M. M. Abdel‐Daim , “Multi‐Target Drug Candidates for Multifactorial Alzheimer's Disease: AChE and NMDAR as Molecular Targets,” Molecular Neurobiology 58 (2021): 281–303. 32935230 10.1007/s12035-020-02116-9

[10] F. Zhang , R. J. Zhong , C. Cheng , S. Li , and W. D. Le , “New Therapeutics Beyond Amyloid‐β and Tau for the Treatment of Alzheimer's Disease,” Acta Pharmacologica Sinica 42 (2021): 1382–1389. 33268824 10.1038/s41401-020-00565-5 PMC8379190

[11] P. Chopade , N. Chopade , Z. Zhao , S. Mitragotri , R. Liao , and V. Chandran Suja , “Alzheimer's and Parkinson's Disease Therapies in the Clinic,” Bioengineering & Translational Medicine 8 (2023): e10367. 36684083 10.1002/btm2.10367 PMC9842041

[12] T. Behl , R. Makkar , A. Sehgal , et al., “Current Trends in Neurodegeneration: Cross Talks Between Oxidative Stress, Cell Death, and Inflammation,” International Journal of Molecular Sciences 22 (2021): 7432. 34299052 10.3390/ijms22147432 PMC8306752

[13] R. Dhapola , S. K. Beura , P. Sharma , S. K. Singh , and D. HariKrishnaReddy , “Oxidative Stress in Alzheimer's Disease: Current Knowledge of Signaling Pathways and Therapeutics,” Molecular Biology Reports 51 (2024): 48. 38165499 10.1007/s11033-023-09021-z

[14] J. Ali , K. Choe , J. S. Park , et al., “The Interplay of Protein Aggregation, Genetics, and Oxidative Stress in Alzheimer's Disease: Role for Natural Antioxidants and Immunotherapeutics,” Antioxidants 13 (2024): 862. 39061930 10.3390/antiox13070862 PMC11274292

[15] A. Asim , M. K. Jastrzębski , and A. A. Kaczor , “Dual Inhibitors of Acetylcholinesterase and Monoamine Oxidase‐B for the Treatment of Alzheimer's Disease,” Molecules 30 (2025): 2975. 40733241 10.3390/molecules30142975 PMC12301006

[16] P. Cruz‐Vicente , L. A. Passarinha , S. Silvestre , and E. Gallardo , “Recent Developments in New Therapeutic Agents Against Alzheimer and Parkinson Diseases: In‐Silico Approaches,” Molecules 26 (2021): 2193–2221. 33920326 10.3390/molecules26082193 PMC8069930

[17] E. Srinivasan , G. Chandrasekhar , P. Chandrasekar , et al., “Alpha‐Synuclein Aggregation in Parkinson's Disease,” Frontiers of Medicine 8 (2021), 10.3389/fmed.2021.736978. PMC8558257 34733860

[18] S. Yi , L. Wang , H. Wang , M. S. Ho , and S. Zhang , “Pathogenesis of α‐Synuclein in Parkinson's Disease: From a Neuron‐Glia Crosstalk Perspective,” International Journal of Molecular Sciences 23 (2022): 14753. 36499080 10.3390/ijms232314753 PMC9739123

[19] Z. D. Zhou , L. X. Yi , D. Q. Wang , T. M. Lim , and E. K. Tan , “Role of Dopamine in the Pathophysiology of Parkinson's Disease,” Translational Neurodegeneration 12 (2023): 44. 37718439 10.1186/s40035-023-00378-6 PMC10506345

[20] H. Juárez Olguín , D. Calderón Guzmán , E. Hernández García , G. Barragán Mejía , and A.‐L. Bulteau , “The Role of Dopamine and Its Dysfunction as a Consequence of Oxidative Stress,” Oxidative Medicine and Cellular Longevity 2016 (2016): 9730467. 26770661 10.1155/2016/9730467 PMC4684895

[21] L. Speranza , U. Di Porzio , D. Viggiano , A. de Donato , and F. Volpicelli , “Dopamine: The Neuromodulator of Long‐Term Synaptic Plasticity, Reward and Movement Control,” Cells 10 (2021): 735. 33810328 10.3390/cells10040735 PMC8066851

[22] S. S. Kang , E. H. Ahn , Z. Zhang , et al., “α‐Synuclein Stimulation of Monoamine Oxidase‐B and Legumain Protease Mediates the Pathology of Parkinson's Disease,” EMBO Journal 37 (2018), 10.15252/embj.201798878. PMC6003650 29769405

[23] G. S. Baweja , S. Gupta , B. Kumar , P. Patel , and V. Asati , “Recent Updates on Structural Insights of MAO‐B Inhibitors: A Review on Target‐Based Approach,” Molecular Diversity 28 (2024): 1823–1845. 36977955 10.1007/s11030-023-10634-6 PMC10047469

[24] L. Sequeira , S. Benfeito , C. Fernandes , et al., “Drug Development for Alzheimer's and Parkinson's Disease: Where Do We Go Now?,” Pharmaceutics 16 (2024): 708. 38931832 10.3390/pharmaceutics16060708 PMC11206728

[25] FDA , FDA Converts Novel Alzheimer's Disease Treatment to Traditional Approval (2023), https://www.fda.gov/news‐events/press‐announcements/fda‐converts‐novel‐alzheimers‐disease‐treatment‐traditional‐approval.

[26] H. Wang , J. Pan , M. Zhang , and Z. Tan , “Re‐Evaluation of the Efficacy and Safety of Anti‐aβ Monoclonal Antibodies (Lecanemab/Donanemab) in the Treatment of Early Alzheimer's Disease,” Frontiers in Pharmacology 16 (2025), 10.3389/fphar.2025.1599048. PMC12040886 40308765

[27] FDA , FDA approves Treatment for Adults with Alzheimer's Disease (2024), https://www.fda.gov/drugs/news‐events‐human‐drugs/fda‐approves‐treatment‐adults‐alzheimers‐disease.

[28] X. Dong‐Chen , C. Yong , X. Yang , S. Chen‐Yu , and P. Li‐Hua , “Signaling Pathways in Parkinson's Disease: Molecular Mechanisms and Therapeutic Interventions,” Signal Transduction and Targeted Therapy 8 (2023): 73. 36810524 10.1038/s41392-023-01353-3 PMC9944326

[29] M. Regensburger , C. W. Ip , Z. Kohl , et al., “Clinical Benefit of MAO‐B and COMT Inhibition in Parkinson's Disease: Practical Considerations,” Journal of Neural Transmission 130 (2023): 847–861. 36964457 10.1007/s00702-023-02623-8 PMC10199833

[30] F. Stocchi , D. Bravi , A. Emmi , and A. Antonini , “Parkinson Disease Therapy: Current Strategies and Future Research Priorities,” Nature Reviews Neurology 20 (2024): 695–707. 39496848 10.1038/s41582-024-01034-x

[31] J. Cohen , A. Mathew , K. D. Dourvetakis , et al., “Recent Research Trends in Neuroinflammatory and Neurodegenerative Disorders,” Cells 13 (2024): 511. 38534355 10.3390/cells13060511 PMC10969521

[32] S. Reardon , “FDA Approves Alzheimer's Drug Lecanemab Amid Safety Concerns,” Nature 613 (2023): 227–228. 36627422 10.1038/d41586-023-00030-3

[33] G. Marucci , M. Buccioni , D. D. Ben , C. Lambertucci , R. Volpini , and F. Amenta , “Efficacy of Acetylcholinesterase Inhibitors in Alzheimer's Disease,” Neuropharmacology 190 (2021): 108352. 33035532 10.1016/j.neuropharm.2020.108352

[34] T. J. Eckroat , D. L. Manross , and S. C. Cowan , “Merged Tacrine‐Based, Multi‐Target‐Directed Acetylcholinesterase Inhibitors 2015–Present: Synthesis and Biological Activity,” International Journal of Molecular Sciences 21 (2020): 5965. 32825138 10.3390/ijms21175965 PMC7504404

[35] Ó.M. Bautista‐Aguilera , L. Ismaili , I. Iriepa , et al., “Tacrines as Therapeutic Agents for Alzheimer's Disease. V. Recent Developments,” Chemical Record 21 (2021): 162–174. 33169934 10.1002/tcr.202000107

[36] S. Mitra , M. Muni , N. J. Shawon , et al., “Tacrine Derivatives in Neurological Disorders: Focus on Molecular Mechanisms and Neurotherapeutic Potential,” Oxidative Medicine and Cellular Longevity 2022 (2022): 7252882. 36035218 10.1155/2022/7252882 PMC9410840

[37] B. Dogga , E. K. Reddy , C. S. Sharanya , et al., “Design, Synthesis and SAR Studies of Novel Tacrine Derivatives as Potent Cholinesterase Inhibitors,” European Journal of Medicinal Chemistry Reports 6 (2022): 100094.

[38] A. Bubley , A. Erofeev , P. Gorelkin , E. Beloglazkina , A. Majouga , and O. Krasnovskaya , “Tacrine‐Based Hybrids: Past, Present, and Future,” International Journal of Molecular Sciences 24 (2023): 1717–1770. 36675233 10.3390/ijms24021717 PMC9863713

[39] P. Jiri , J. Daniel , and K. Kamil , “Possible Role of Hydroxylated Metabolites of Tacrine in Drug Toxicity and Therapy of Alzheimers Disease,” Current Drug Metabolism 9 (2008): 332–335. 18473751 10.2174/138920008784220619

[40] M. Novak , M. Vajrychova , S. Koutsilieri , et al., “Tacrine First‐Phase Biotransformation and Associated Hepatotoxicity: A Possible Way to Avoid Quinone Methide Formation,” ACS Chemical Biology 18 (2023): 1993–2002. 37622522 10.1021/acschembio.3c00219

[41] S. Fares , W. M. El Husseiny , K. B. Selim , and M. A. M. Massoud , “Modified Tacrine Derivatives as Multi‐Target‐Directed Ligands for the Treatment of Alzheimer's Disease: Synthesis, Biological Evaluation, and Molecular Modeling Study,” ACS Omega 8 (2023): 26012–26034. 37521639 10.1021/acsomega.3c02051 PMC10373466

[42] M. Chvojkova , D. Kolar , K. Kovacova , et al., “Pro‐Cognitive Effects of Dual Tacrine Derivatives Acting as Cholinesterase Inhibitors and NMDA Receptor Antagonists,” Biomedicine & Pharmacotherapy 176 (2024): 116821. 38823278 10.1016/j.biopha.2024.116821

[43] V. Yadav , J. Reang , V. Sharma , et al., “Quinoline‐Derivatives as Privileged Scaffolds for Medicinal and Pharmaceutical Chemists: A Comprehensive Review,” Chemical Biology & Drug Design 100 (2022): 389–418. 35712793 10.1111/cbdd.14099

[44] M. S. S. Chauhan , T. Umar , and M. K. Aulakh , “Quinolines: Privileged Scaffolds for Developing New Anti‐Neurodegenerative Agents,” ChemistrySelect 8 (2023): e202204960.

[45] I. A. Bala , O. F. Al Sharif , A. M. Asiri , and R. M. El‐Shishtawy , “Quinoline: A Versatile Bioactive Scaffold and Its Molecular Hybridization,” Results in Chemistry 7 (2024): 101529.

[46] R. B. Morais , M. d. Sacramento , C. Scimmi , et al., “DFT‐Based Elucidation and Evaluation of Selenium‐Modified Tacrine Derivatives: Theoretical and Physicochemical Insights for Alzheimer's Disease Therapy,” Molecules 30 (2025): 2553. 40572521 10.3390/molecules30122553 PMC12196396

[47] M. M. Reddy , A. J , E. K. Reddy , S. Anwar , and S. C , “Synthesis, Docking, In Vitro and in Silico Investigations of Novel Tacrine Derivatives as Acetylcholinesterase Inhibitors,” Organic & Biomolecular Chemistry 23 (2025): 6773–6784. 40586736 10.1039/d5ob00785b

[48] B. Svobodova , Z. Moravcova , A. Misiachna , et al., “Novel Tacrine‐Based Multi‐Target Directed Ligands: Enhancing Cholinesterase Inhibition, NMDA Receptor Antagonism, and CNS Bioavailability for Alzheimer's Disease Treatment,” European Journal of Medicinal Chemistry 292 (2025): 117678. 40288120 10.1016/j.ejmech.2025.117678

[49] E. G. Guzmán‐López , M. Reina , L. F. Hernández‐Ayala , and A. Galano , “Rational Design of Multifunctional Ferulic Acid Derivatives Aimed for Alzheimer's and Parkinson's Diseases,” Antioxidants 12 (2023): 1256. 37371986 10.3390/antiox12061256 PMC10295297

[50] E. G. Guzman‐Lopez , M. Reina , A. Perez‐Gonzalez , et al., “CADMA‐Chem: A Computational Protocol Based on Chemical Properties Aimed to Design Multifunctional Antioxidants,” International Journal of Molecular Sciences 23 (2022): 13246–13272. 36362034 10.3390/ijms232113246 PMC9658414

[51] M. Reina , E. G. Guzmán‐López , and A. Galano , “Computational Design of Rasagiline Derivatives: Searching for Enhanced Antioxidant Capability,” International Journal of Quantum Chemistry 123 (2023): e27011.

[52] A. Pérez‐González , R. Castañeda‐Arriaga , E. G. Guzmán‐López , L. F. Hernández‐Ayala , and A. Galano , “Chalcone Derivatives With a High Potential as Multifunctional Antioxidant Neuroprotectors,” ACS Omega 7 (2022): 38254–38268. 36340167 10.1021/acsomega.2c05518 PMC9631883

[53] L. F. Hernández‐Ayala , E. G. Guzmán‐López , and A. Galano , “Quinoline Derivatives: Promising Antioxidants With Neuroprotective Potential,” Antioxidants 12 (2023): 1853. 37891932 10.3390/antiox12101853 PMC10604020

[54] B. Morales‐Garcia , A. Pérez‐González , J. R. Álvarez‐Idaboy , and A. Galano , “Computer‐Aided Design of Caffeic Acid Derivatives: Free Radical Scavenging Activity and Reaction Force,” Journal of Molecular Modeling 31 (2025): 1–14. 10.1007/s00894-024-06226-2 PMC11680653 39729167

[55] M. Prejanò , I. Romeo , L. Felipe Hernández‐Ayala , et al., “Evaluating Quinolines: Molecular Dynamics Approach to Assess Their Potential as Acetylcholinesterase Inhibitors for Alzheimer's Disease,” ChemPhysChem 26 (2025): e202400653. 39301943 10.1002/cphc.202400653 PMC11747580

[56] M. Spiegel , “Theoretical Insights Into the Oxidative Stress‐Relieving Properties of Pinocembrin—An Isolated Flavonoid From Honey and Propolis,” Journal of Physical Chemistry B 127 (2023): 8769–8779. 37816048 10.1021/acs.jpcb.3c03545 PMC10591471

[57] M. Spiegel , “Unveiling the Antioxidative Potential of Galangin: Complete and Detailed Mechanistic Insights Through Density Functional Theory Studies,” Journal of Organic Chemistry 89 (2024): 8676–8690. 38861646 10.1021/acs.joc.4c00611 PMC11197094

[58] W. E. Prasetyo , V. Kurniansyah , M. Firdaus , et al., “Free Radical Scavenging Behaviour of the Plant‐Beneficial Secondary Metabolite 2,4‐Diacetylphloroglucinol Derivatives Under Physiological Conditions: A Joint Experimental and Theoretical Study,” Journal of Molecular Structure 1302 (2024): 137498.

[59] M. Spiegel , “Fisetin as a Blueprint for Senotherapeutic Agents – Elucidating Geroprotective and Senolytic Properties With Molecular Modeling,” Chemistry – A European Journal 31 (2025): e202403755. 39688310 10.1002/chem.202403755 PMC11914956

[60] A. Amić , D. Mastiľák Cagardová , and Ž. Milanović , “Theoretical Study of Antioxidant and Prooxidant Potency of Protocatechuic Aldehyde,” International Journal of Molecular Sciences 26 (2025): 404. 39796260 10.3390/ijms26010404 PMC11721355

[61] Ž. Milanović , S. Jeremić , J.Đ. Jovanović , M. Antonijević , and K. Milisavljević , “Rational Computational Design of Bergaptol Derivatives: Antioxidant Potential and CYP3A4 Inhibitory Activity,” ChemistrySelect 10 (2025): e202500375.

[62] E. A. Tejeda‐Medina , C. Espinoza , A. V. Jerezano , and M. E. Medina , “Insight From the Theory of the Multi‐Target Antioxidant Potency of Toluquinol,” Journal of Physical Chemistry B 129 (2025): 8464–8472. 40767958 10.1021/acs.jpcb.5c01589

[63] K. Milisavljević and Ž. Milanović , “Multifunctional Tetrahydrocannabinol Derivatives as Potential Antioxidant Neuroprotectors: Computer‐Aided Design and Analysis,” Journal of Computational Biophysics and Chemistry 25 (2026): 1815–1837.

[64] O. Benek , J. Korabecny , and O. Soukup , “A Perspective on Multi‐Target Drugs for Alzheimer's Disease,” Trends in Pharmacological Sciences 41 (2020): 434–445. 32448557 10.1016/j.tips.2020.04.008

[65] E. G. Guzman‐Lopez and A. Galano , Smile‐it, 1.0, UAM‐Iztapalapa, CDMX, 2024, https://agalano.com/research/apps/desktop‐apps/.

[66] E. G. Guzman‐Lopez and A. Galano , CADMA‐Py, 1.0, UAM‐Iztapalapa, CDMX, 2024, https://agalano.com/research/apps/desktop‐apps/.

[67] C. A. Lipinski , F. Lombardo , B. W. Dominy , and P. J. Feeney , “Experimental and Computational Approaches to Estimate Solubility and Permeability in Drug Discovery and Development Settings,” Advanced Drug Delivery Reviews 23 (1997): 3–25. 10.1016/s0169-409x(00)00129-0 11259830

[68] A. K. Ghose , V. N. Viswanadhan , and J. J. Wendoloski , “A Knowledge‐Based Approach in Designing Combinatorial or Medicinal Chemistry Libraries for Drug Discovery. 1. A Qualitative and Quantitative Characterization of Known Drug Databases,” Journal of Combinatorial Chemistry 1 (1999): 55–68. 10746014 10.1021/cc9800071

[69] W. J. Egan , K. M. Merz , and J. J. Baldwin , “Prediction of Drug Absorption Using Multivariate Statistics,” Journal of Medicinal Chemistry 43 (2000): 3867–3877. 11052792 10.1021/jm000292e

[70] I. Muegge , S. L. Heald , and D. Brittelli , “Simple Selection Criteria for Drug‐Like Chemical Matter,” Journal of Medicinal Chemistry 44 (2001): 1841–1846. 11384230 10.1021/jm015507e

[71] D. F. Veber , S. R. Johnson , H.‐Y. Cheng , B. R. Smith , K. W. Ward , and K. D. Kopple , “Molecular Properties that Influence the Oral Bioavailability of Drug Candidates,” Journal of Medicinal Chemistry 45 (2002): 2615–2623. 12036371 10.1021/jm020017n

[72] Y. Gu , Z. Yu , Y. Wang , et al., “admetSAR3.0: A Comprehensive Platform for Exploration, Prediction and Optimization of Chemical ADMET Properties,” Nucleic Acids Research 52 (2024): W432–W438. 38647076 10.1093/nar/gkae298 PMC11223829

[73] L. Fu , S. Shi , J. Yi , et al., “ADMETlab 3.0: An Updated Comprehensive Online ADMET Prediction Platform Enhanced With Broader Coverage, Improved Performance, API Functionality and Decision Support,” Nucleic Acids Research 52 (2024): W422–W431. 38572755 10.1093/nar/gkae236 PMC11223840

[74] P. Banerjee , E. Kemmler , M. Dunkel , and R. Preissner , “ProTox 3.0: A Webserver for the Prediction of Toxicity of Chemicals,” Nucleic Acids Research 52 (2024): W513–W520. 38647086 10.1093/nar/gkae303 PMC11223834

[75] R. S. Tieghi , M. Rath , J. T. Moreira‐Filho , et al., “DeTox: An in‐Silico Alternative to Animal Testing for Predicting Developmental Toxicity Potential,” Environ Health Perspect (2025), 10.1289/ehp15307. 40388119

[76] S. Seal , D. Williams , L. Hosseini‐Gerami , et al., “Improved Detection of Drug‐Induced Liver Injury by Integrating Predicted In Vivo and In Vitro Data,” Chemical Research in Toxicology 37 (2024): 1290–1305. 38981058 10.1021/acs.chemrestox.4c00015 PMC11337212

[77] J. Han , W. Zhung , I. Jang , et al., “HepatoToxicity Portal (HTP): An Integrated Database of Drug‐Induced Hepatotoxicity Knowledgebase and Graph Neural Network‐Based Prediction Model,” Journal of Cheminformatics 17 (2025): 48. 40200282 10.1186/s13321-025-00992-8 PMC11980326

[78] N. Kochev , S. Avramova , P. Angelov , and N. Jeliazkova , “Computational Prediction of Synthetic Accessibility of Organic Molecules With Ambit‐Synthetic Accessibility Tool,” Organic Chemistry: An Indian Journal 14 (2018): 123.

[79] Y. Zhao , N. E. Schultz , and D. G. Truhlar , “Design of Density Functionals by Combining the Method of Constraint Satisfaction With Parametrization for Thermochemistry, Thermochemical Kinetics, and Noncovalent Interactions,” Journal of Chemical Theory and Computation 2 (2006): 364–382. 26626525 10.1021/ct0502763

[80] A. Galano and J. R. Alvarez‐Idaboy , “Kinetics of Radical‐Molecule Reactions in Aqueous Solution: A Benchmark Study of the Performance of Density Functional Methods,” Journal of Computational Chemistry 35 (2014): 2019–2026. 25142611 10.1002/jcc.23715

[81] A. Galano , “Free Radicals Induced Oxidative Stress at a Molecular Level: The Current Status, Challenges and Perspectives of Computational Chemistry Based Protocols,” Journal of the Mexican Chemical Society 59 (2015): 231–262.

[82] Y. Zhao and D. G. Truhlar , “How Well Can New‐Generation Density Functionals Describe the Energetics of Bond‐Dissociation Reactions Producing Radicals?,” Journal of Physical Chemistry A 112 (2008): 1095–1099. 18211046 10.1021/jp7109127

[83] A. Galano , A. Pérez‐González , R. Castañeda‐Arriaga , et al., “Empirically Fitted Parameters for Calculating pKa Values With Small Deviations From Experiments Using a Simple Computational Strategy,” Journal of Chemical Information and Modeling 56 (2016): 1714–1724. 27585285 10.1021/acs.jcim.6b00310

[84] A. M. Rebollar‐Zepeda and A. Galano , “First Principles Calculations of pKa Values of Amines in Aqueous Solution: Application to Neurotransmitters,” International Journal of Quantum Chemistry 112 (2012): 3449–3460.

[85] A. Pérez‐González , R. Castañeda‐Arriaga , B. Verastegui , M. Carreón‐González , J. R. Alvarez‐Idaboy , and A. Galano , “Estimation of Empirically Fitted Parameters for Calculating pKa Values of Thiols in a Fast and Reliable Way,” Theoretical Chemistry Accounts 137 (2018): 1–10.

[86] A. V. Marenich , C. J. Cramer , and D. G. Truhlar , “Universal Solvation Model Based on Solute Electron Density and on a Continuum Model of the Solvent Defined by the Bulk Dielectric Constant and Atomic Surface Tensions,” Journal of Physical Chemistry B 113 (2009): 6378–6396. 19366259 10.1021/jp810292n

[87] M. J. Frisch , G. W. Trucks , H. B. Schlegel , et al., Gaussian 09, Rev. D.01 (Gaussian Inc, 2013), http://gaussian.com/.

[88] E. Dzib , J. L. Cabellos , F. Ortíz‐Chi , S. Pan , A. Galano , and G. Merino , “Eyringpy: A Program for Computing Rate Constants in the Gas Phase and in Solution,” International Journal of Quantum Chemistry 119 (2019): e25686.

[89] Marvin, Chemaxon, Budapest , 2024, https://playground.calculators.cxn.io/.

[90] H. M. Berman , J. Westbrook , Z. Feng , et al., “The Protein Data Bank,” Nucleic Acids Research 28 (2000): 235–242. 10592235 10.1093/nar/28.1.235 PMC102472

[91] J. Cheung , M. J. Rudolph , F. Burshteyn , et al., “Structures of Human Acetylcholinesterase in Complex With Pharmacologically Important Ligands,” Journal of Medicinal Chemistry 55 (2012): 10282–10286. 23035744 10.1021/jm300871x

[92] T.‐H. Chou , M. Epstein , K. Michalski , E. Fine , P. C. Biggin , and H. Furukawa , “Structural Insights Into Binding of Therapeutic Channel Blockers in NMDA Receptors,” Nature Structural & Molecular Biology 29 (2022): 507–518. 10.1038/s41594-022-00772-0 PMC10075384 35637422

[93] M. Ellermann , C. Lerner , G. Burgy , et al., “Catechol‐O‐Methyltransferase in Complex With Substituted 3′‐Deoxyribose Bisubstrate Inhibitors,” Biological Crystallography 68 (2012): 253–260. 22349227 10.1107/S0907444912001138

[94] C. Binda , J. Wang , L. Pisani , et al., “Structures of Human Monoamine Oxidase B Complexes With Selective Noncovalent Inhibitors: Safinamide and Coumarin Analogs,” Journal of Medicinal Chemistry 50 (2007): 5848–5852. 17915852 10.1021/jm070677y

[95] B. Webb and A. Sali , “Comparative Protein Structure Modeling Using MODELLER,” Current Protocols in Bioinformatics 54 (2016): 5.6.1–5.6. 37. 10.1002/cpbi.3 PMC5031415 27322406

[96] T. Schwede , J. Kopp , N. Guex , and M. C. Peitsch , “SWISS‐MODEL: An Automated Protein Homology‐Modeling Server,” Nucleic Acids Research 31 (2003): 3381–3385. 12824332 10.1093/nar/gkg520 PMC168927

[97] G. M. Morris , R. Huey , W. Lindstrom , et al., “AutoDock4 and AutoDockTools4: Automated Docking With Selective Receptor Flexibility,” Journal of Computational Chemistry 30 (2009): 2785–2791. 19399780 10.1002/jcc.21256 PMC2760638

[98] J. Eberhardt , D. Santos‐Martins , A. F. Tillack , and S. Forli , “AutoDock Vina 1.2.0: New Docking Methods, Expanded Force Field, and Python Bindings,” Journal of Chemical Information and Modeling 61 (2021): 3891–3898. 34278794 10.1021/acs.jcim.1c00203 PMC10683950

[99] O. Trott and A. J. Olson , “AutoDock Vina: Improving the Speed and Accuracy of Docking With a New Scoring Function, Efficient Optimization, and Multithreading,” Journal of Computational Chemistry 31 (2010): 455–461. 19499576 10.1002/jcc.21334 PMC3041641

[100] L. F. Hernández‐Ayala , R.‐J. Reiter , and A. Galano , “Melatonin and Related Compounds as Enzymatic Antioxidants: A Comprehensive Theoretical Study,” BIOCELL 8 (2026): 1–39.

[101] BIOVIA Discovery Studio , Dassault Systèmes, San Diego, 2025, https://discover.3ds.com/discovery‐studio‐visualizer‐download.

[102] E. F. Pettersen , T. D. Goddard , C. C. Huang , et al., “UCSF Chimera—a Visualization System for Exploratory Research and Analysis,” Journal of Computational Chemistry 25 (2004): 1605–1612. 15264254 10.1002/jcc.20084

[103] S. Jo , T. Kim , V. G. Iyer , and W. Im , “CHARMM‐GUI: A Web‐Based Graphical User Interface for CHARMM,” Journal of Computational Chemistry 29 (2008): 1859–1865. 18351591 10.1002/jcc.20945

[104] J. Huang and A. D. MacKerell Jr , “CHARMM36 All‐Atom Additive Protein Force Field: Validation Based on Comparison to NMR Data,” Journal of Computational Chemistry 34 (2013): 2135–2145. 23832629 10.1002/jcc.23354 PMC3800559

[105] M. J. Abraham , T. Murtola , R. Schulz , et al., “GROMACS: High Performance Molecular Simulations Through Multi‐Level Parallelism From Laptops to Supercomputers,” SoftwareX 1‐2 (2015): 19–25.

[106] W. L. Jorgensen , J. Chandrasekhar , J. D. Madura , R. W. Impey , and M. L. Klein , “Comparison of Simple Potential Functions for Simulating Liquid Water,” The Journal of Chemical Physics 79 (1983): 926–935.

[107] P. T. Männistö , T. Keränen , K. J. Reinikainen , A. Hanttu , and P. Pollesello , “The Catechol O‐Methyltransferase Inhibitor Entacapone in the Treatment of Parkinson's Disease: Personal Reflections on a First‐in‐Class Drug Development Programme 40 Years on,” Neurology and Therapy 13 (2024): 1039–1054. 38809484 10.1007/s40120-024-00629-2 PMC11263458

[108] G. Bussi , D. Donadio , and M. Parrinello , “Canonical Sampling Through Velocity Rescaling,” The Journal of Chemical Physics 126 (2007): 014101. 17212484 10.1063/1.2408420

[109] M. Bernetti and G. Bussi , “Pressure Control Using Stochastic Cell Rescaling,” The Journal of Chemical Physics 153 (2020): 114107. 32962386 10.1063/5.0020514

[110] B. Hess , H. Bekker , H. J. C. Berendsen , and J. G. E. M. Fraaije , “LINCS: A Linear Constraint Solver for Molecular Simulations,” Journal of Computational Chemistry 18 (1997): 1463–1472.

[111] A. Galano , “Antioxidant Activity at the Molecular Level: Exploring Ways of Action and Computational Tools to Investigate Them,” Chemical Science 16 (2025): 19570–19593. 41111544 10.1039/d5sc05463j PMC12529800

[112] A. Galano , “Antioxidants: The Chemical Complexity Behind a Simple Word,” Accounts of Chemical Research 58 (2025): 3481–3493. 40931004 10.1021/acs.accounts.5c00552 PMC12676661

[113] C. Binda , M. Aldeco , W. J. Geldenhuys , M. Tortorici , A. Mattevi , and D. E. Edmondson , “Molecular Insights Into Human Monoamine Oxidase B Inhibition by the Glitazone Antidiabetes Drugs,” ACS Medicinal Chemistry Letters 3 (2012): 39–42. 10.1021/ml200196p PMC3263826 22282722

[114] C. Da and D. Kireev , “Structural Protein–Ligand Interaction Fingerprints (SPLIF) for Structure‐Based Virtual Screening: Method and Benchmark Study,” Journal of Chemical Information and Modeling 54 (2014): 2555–2561. 25116840 10.1021/ci500319f PMC4170813

[115] G. Marcou and D. Rognan , “Optimizing Fragment and Scaffold Docking by use of Molecular Interaction Fingerprints,” Journal of Chemical Information and Modeling 47 (2007): 195–207. 17238265 10.1021/ci600342e

[116] Z. Deng , C. Chuaqui , and J. Singh , “Structural Interaction Fingerprint (SIFt): A Novel Method for Analyzing Three‐Dimensional Protein‐Ligand Binding Interactions,” Journal of Medicinal Chemistry 47 (2004): 337–344. 14711306 10.1021/jm030331x

[117] S. Y. Son , J. Ma , Y. Kondou , M. Yoshimura , E. Yamashita , and T. Tsukihara , “Structure of Human Monoamine Oxidase a at 2.2‐a Resolution: the Control of Opening the Entry for Substrates/Inhibitors,” Proceedings of the National Academy of Sciences of the United States of America 105 (2008): 5739–5744. 18391214 10.1073/pnas.0710626105 PMC2311356

[118] M. B. Youdim , D. Edmondson , and K. F. Tipton , “The Therapeutic Potential of Monoamine Oxidase Inhibitors,” Nature Reviews Neuroscience 7 (2006): 295–309. 16552415 10.1038/nrn1883

[119] J. P. Finberg , “Update on the Pharmacology of Selective Inhibitors of MAO‐a and MAO‐B: Focus on Modulation of CNS Monoamine Neurotransmitter Release,” Pharmacology & Therapeutics 143 (2014): 133–152. 24607445 10.1016/j.pharmthera.2014.02.010

[120] S. Carradori , M. C. Gidaro , A. Petzer , et al., “Inhibition of Human Monoamine Oxidase: Biological and Molecular Modeling Studies on Selected Natural Flavonoids,” Journal of Agricultural and Food Chemistry 64 (2016): 9004–9011. 27933876 10.1021/acs.jafc.6b03529

[121] M. Harel , I. Schalk , L. Ehret‐Sabatier , et al., “Quaternary Ligand Binding to Aromatic Residues in the Active‐Site Gorge of Acetylcholinesterase,” Proceedings of the National Academy of Sciences 90 (1993): 9031–9035. 10.1073/pnas.90.19.9031 PMC47495 8415649

[122] M. B. Colović , D. Z. Krstić , T. D. Lazarević‐Pašti , A. M. Bondžić , and V. M. Vasić , “Acetylcholinesterase Inhibitors: Pharmacology and Toxicology,” Current Neuropharmacology 11 (2013): 315–335. 24179466 10.2174/1570159X11311030006 PMC3648782

[123] N. C. Inestrosa , A. Alvarez , C. A. Pérez , et al., “Acetylcholinesterase Accelerates Assembly of Amyloid‐Beta‐Peptides Into Alzheimer's Fibrils: Possible Role of the Peripheral Site of the Enzyme,” Neuron 16 (1996): 881–891. 8608006 10.1016/s0896-6273(00)80108-7

[124] M. J. Bonifácio , P. N. Palma , L. Almeida , and P. Soares‐da‐Silva , “Catechol‐O‐Methyltransferase and Its Inhibitors in Parkinson's Disease,” CNS Drug Reviews 13 (2007): 352–379. 17894650 10.1111/j.1527-3458.2007.00020.x PMC6494163

[125] P. T. Männistö and S. Kaakkola , “Catechol‐O‐Methyltransferase (COMT): Biochemistry, Molecular Biology, Pharmacology, and Clinical Efficacy of the New Selective COMT Inhibitors,” Pharmacological Reviews 51 (1999): 593–628. 10581325

[126] S. A. Lipton and H. S. Chen , “Paradigm Shift in NMDA Receptor Drug Development,” Expert Opinion on Therapeutic Targets 9 (2005): 427–429. 15948664 10.1517/14728222.9.3.427

