# Supplementary material for: Rational Design of Multifunctional Tacrine Derivatives as Candidates for the Treatment of Alzheimer and Parkinson Diseases
Source: ChemMedChem. 2026 Aug 10;21(15):e70429. doi: 10.1002/cmdc.70429 (PMC13456978; doi:10.1002/cmdc.70429)
Supplement: Supplementary file 1 [file CMDC-21-e70429-s001.pdf]

Rational Design of Multifunctional Tacrine as Candidates for the treatment of Alzheimer and Parkinson Diseases

Eugenia Dzib,[1] Luis Felipe Hernández-Ayala,[1] Silvana Silva-Aguirre,[2] and Annia Galano[1,*]

[1] *Universidad Autónoma Metropolitana, Unidad Iztapalapa, División de Ciencias Básicas e Ingeniería, Departamento de Química, Ferrocarril San Rafael Atlixco 186, Col. Leyes de Reforma 1a Sección, Alcaldía Iztapalapa, 09130, Ciudad de México, México*

[2] *Departamento de Física Aplicada, Centro de Investigación y de Estudios Avanzados, Unidad Mérida, km 6 Antigua Carretera a Progreso, Apdo. Postal 73, Cordemex, 97310 Mérida, Yucatán, México*

Correspondence to Annia Galano (e-mail: agal@xanum.uam.mx)

CONTENIDO

Text S1. Selection, multi-target, and similarity interaction scores. 3

Table S1. Different programs used to assess the toxicity parameters: Oral rat 50% lethal dose (LD_{50}), Ames mutagenicity (M), Developmental toxicity (DT), and Drug-Induced Liver Injury (DILI). 7

Table S6. Values of the ADME properties of the reference set for combinations 1, 2, and 3: molecular weight (MW in g mol^{-1}), water/octanol partition coefficient (logP), molar refractivity (MR in m^3 mol^{-1}), number of heavy atoms (XAt), number of H-bond acceptors (HBA), number of H-bond donors (HBD), rotatable bonds (RB), and topological polar surface area (PSA in Å^2); toxicity descriptors: Oral rat 50% lethal dose (LD_{50} in mg kg^{-1}), Ames mutagenicity (M), and Developmental toxicity (DT); and the synthetic accessibility (SA). 9

Table S7. Values of Drug-Induced Liver Injury (DILI) of tacrine and its 10 best-scored derivatives. 12

Table S8. First ionization energy (IE, eV) and bond dissociation energies (BDE, kcal mol^{-1}) for tacrine (TAC) and the selected subset of derivatives. The *q* represents the charge of the acid-base species. 13

Table S9. Binding energy (kcal mol^{-1}) and weighted binding energy (kcal mol^{-1}) of the acid-base species for tacrine and the selected subset of derivatives. 15

Table S10. Interactions of tacrine and the subset of selected derivatives with the proteins used to study the interaction pattern. 16
Table S11. Interaction-matching parameters used for the calculation. 17
Figure S1. Deprotonation routes for tacrine and the derivatives selected as the most promising, due to their drug-like behavior. 18
Figure S2. Molar fractions for tacrine and the subset of derivatives selected as the most promising, due to their drug-like behavior. The vertical dotted line corresponds to the molar fractions at pH = 7.4. 19
Figure S3. Multi-target efficiency plot of tacrine and its derivatives relative to the reference drugs. 20
Figure S4. MD-100ns plots for the complex [MAO-B:TAC-678] complex....................... 21
Figure S5. MD-100ns plots for the complex [AChE:TAC-674] complex. 22

Text S1. Selection, multi-target, and similarity interaction scores.

The selection score S^s (Eq. 1) is designed to identify the derivatives with the best drug-like behavior. It gives a weight to each derivative depending on whether they meet the physicochemical parameters necessary to evaluate ADME properties (S^{ADME}) (Eq. 2), as well as toxicity (S^T) (Eq. 11), and synthetic accessibility (S^{SA}) (Eq. 15).

$$S^s = (0.4 \times S^{ADME}) + (0.4 \times S^T) + (0.2 \times S^{SA}), \quad (1)$$

$$S^{ADME} = \frac{S^{log\,P} + S^{HBD} + S^{HBA} + S^{MW} + S^{MR} + S^{XAt} + S^{RB} + S^{PSA}}{8}, \quad (2)$$

$$S^{log\,P} = \begin{cases} 1, if -0.4 \leq log\,P \leq 5.0 \\ 0, otherwise \end{cases} \quad (3)$$

$$S^{HBD} = \begin{cases} 1, if\ HBD \leq 5 \\ 0, otherwise \end{cases} \quad (4)$$

$$S^{HBA} = \begin{cases} 1, if\ HBA \leq 10 \\ 0, otherwise \end{cases} \quad (5)$$

$$S^{MW} = \begin{cases} 1, if\ 200 \leq MW \leq 480 \\ 0, otherwise \end{cases} \quad (6)$$

$$S^{MR} = \begin{cases} 1, if\ 40 \leq MR \leq 130 \\ 0, otherwise \end{cases} \quad (7)$$

$$S^{XAt} = \begin{cases} 1, if\ 20 \leq XAt \leq 70 \\ 0, otherwise \end{cases} \quad (8)$$

$$S^{RB} = \begin{cases} 1, if\ RB \leq 10 \\ 0, otherwise \end{cases} \quad (9)$$

$$S^{PSA} = \begin{cases} 1, if\ PSA \leq 130 \\ 0, otherwise \end{cases} \tag{10}$$

$$S^{T} = \frac{S^{LD_{50}} + S^{M} + S^{DT}}{3}, \tag{11}$$

$$S^{LD_{50}} = 1 + log\left(\frac{1 + LD_{50}^{dTAC}}{1 + LD_{50}^{Ref\ Set}}\right), \tag{12}$$

$$S^{M} = 1 - log\left(\frac{1 + M^{dTAC}}{1 + M^{Ref\ Set}}\right), \tag{13}$$

$$S^{DT} = 1 - log\left(\frac{1 + DT^{dTAC}}{1 + DT^{Ref\ Set}}\right), \tag{14}$$

$$S^{SA} = \frac{SA^{dTAC}}{SA^{Ref\ Set}}, \tag{15}$$

where $dTAC$ is the tacrine derivative.

The weighted binding energy ΔG_B^W is given by:

$$\Delta G_B^W = \sum_{i}^{n} \Delta G_{B,dTAC}^{protein} \times M_{f(q_i)}, \tag{16}$$

where n is the number of acid-base specie and q_i is the charge of the ith acid-base specie.

The multi-target score S^{MT} evaluates the capacity of the tacrine derivatives to bind to the proteins AChE, NMDAr, COMT, and MAO-B respect to the natural substrates (acetylcholine, glutamate, dopamine, and phenylethylamine).

$$S^{MT} = \frac{\Delta G_{B,dTAC}^{AChE}}{\Delta G_{B,acetylcholine}^{AChE}} + \frac{\Delta G_{B,dTAC}^{NMDAr}}{\Delta G_{B,glutamate}^{NMDA}} + \frac{\Delta G_{B,dTAC}^{COMT}}{\Delta G_{B,dopamine}^{COMT}} + \frac{\Delta G_{B,dTAC}^{MAO-B}}{\Delta G_{B,phenylethylamine}^{MAO-B}} \tag{17}$$

In addition to binding affinities in the protein-ligand complex, it is essential to analyze the conformation and type of interactions contributing to the complex stability to elucidate the inhibition mechanism suggested by the molecular docking simulations. To this end, we developed a parameter that allows comparing the interaction patterns of the studied compounds with those of the corresponding natural substrate or the reference ligands. This parameter is called the Similarity Interaction Score (S_{SI}) and is defined as follows:

$$S_{SI} = \frac{N_{MR} + N_{MI}}{2N_{RR}}, \tag{18}$$

where N_{MR} is the number of residues present in both the compound and the reference ligand, N_{MI} is the number of exact matches in the interactions type (H-bond, π-forces, etc.) on those residues, and N_{RR} is the total number of residues involved in the interaction between the protein and the reference ligand. This index is normalized to have a value of 1 for an exact match and 0 for no match. It is expected to allow for filtering out candidates that strongly bind to the same site of the reference compound, but with the interaction involving different residues or type of interaction. In this work, the index is used solely as an auxiliary descriptor to assess the similarity of interaction patterns. It should not be interpreted as an independent predictor of binding affinity, enzyme inhibition, or biological activity. Its purpose is to provide a transparent and reproducible way to compare whether candidate compounds replicate specific interaction characteristics of natural substrates or reference drugs within the same protein system.

To detect the most promising candidates an internal reference value similarity index (S_{SI}^{Ref}) value was established as:

$$S_{SI}^{Ref} = \frac{S_{SI}^{AChE} + S_{SI}^{NMDAr} + S_{SI}^{COMT} + S_{SI}^{MAO-B}}{4} \quad (19)$$

where S_{SI}^{enzyme} is the score obtained by comparing the protein-ligand interaction pattern of a given compound with that of the reference drug.

This value should not be interpreted as a universal threshold or as an independently validated cut-off for biological activity. It is used only to contextualize S_{SI} values within the present set of proteins, reference ligands, and tacrine derivatives.

Table S1. Different programs used to assess the toxicity parameters: Oral rat 50% lethal dose (LD_{50}), Ames mutagenicity (M), Developmental toxicity (DT), and Drug-Induced Liver Injury (DILI).

Software	LD_{50}		M		DT		DILI	
	In the domain	Data type	In the domain	Data type	In the domain	Data type	In the domain	Data type
admetSAR3.0	Yes	Quantitative (Float in $-\log_{10}$ (mg kg^{-1}))	Yes	Quantitative (Probability) Qualitative (Positive/Negative)	N/A	N/A	Yes	Quantitative (Probability) Qualitative (Positive/Negative)
ADMETlab3.0	Yes	Quantitative (Probability) Qualitative (Excellent/Medium/Poor)	Yes	Quantitative (Probability) Qualitative (Excellent/Medium/Poor)	N/A	N/A	Yes	Quantitative (Probability) Qualitative (Excellent/Medium/Poor)
ProTox3.0	No[a]	Quantitative (Float in mg kg^{-1})	Yes	Quantitative (Probability) Qualitative (Active/Inactive)	N/A	N/A	Yes	Quantitative (Probability) Qualitative (Active/Inactive)
TEST5.1.2	No[b]	Quantitative (Float in mg kg^{-1})	No[b]	Quantitative (Probability)	No[b]	Quantitative (Probability)	N/A	N/A
DeTox	N/A	N/A	N/A	N/A	Yes	Quantitative (Probability) Qualitative	N/A	N/A

						(Toxic/Non-toxic)		
pkCSM	Unknown	Quantitative (Float in mg kg^{-1})[c]	Unknown	Qualitative (Yes/No)	N/A	N/A	N/A	N/A
VEGA1.2.6	No[d]	Quantitative (Float in mg kg^{-1})	No[d]	Qualitative (Yes/No)	No[d]	Qualitative (Yes/No)	No[d]	Qualitative (Yes/No)
DILI-predictor	N/A	N/A	N/A	N/A	N/A	N/A	Yes	Quantitative (Probability) Qualitative (Positive/Negative)
HTP	N/A	N/A	N/A	N/A	N/A	N/A	Yes	Quantitative (Probability) Qualitative (Positive/Negative)

[a] It yielded two LD_{50} values for the 93% of the tacrine derivatives. [b] It did not yield the LD_{50}, M, and DT for 90.0%, 55.5%, and 7.4%, respectively, of the tacrine derivatives. [c] Units are incorrect. However, it was impossible to contact the authors of this software. [d] It did not yield the LD_{50}, M, DT, and DILI for 90.0-100% of the tacrine derivatives. N/A means the given software do not offer the calculation of a given property.

Table S6. Values of the ADME properties of the reference set for combinations 1, 2, and 3: molecular weight (MW in g mol^{-1}), water/octanol partition coefficient (logP), molar refractivity (MR in m^3 mol^{-1}), number of heavy atoms (XAt), number of H-bond acceptors (HBA), number of H-bond donors (HBD), rotatable bonds (RB), and topological polar surface area (PSA in $Å^2$); toxicity descriptors: Oral rat 50% lethal dose (LD_{50} in mg kg^{-1}), Ames mutagenicity (M), and Developmental toxicity (DT); and the synthetic accessibility (SA).

Compound	MW	logP	MR	XAt	HBA	HBD	RB	PSA	LD_{50}	M			DT	SA
										1	2	3		
Fingolimod	307.48	3.20	92.93	22	3	4	12	66.48	1308.53	0.09	0.20	0.84	0.63	86.39
Trofinetide	315.33	-1.24	74.84	22	9	5	7	150.03	4773.19	0.13	0.07	0.86	0.57	80.87
Donepezil	379.50	4.36	110.13	28	4	0	6	38.77	393.66	0.31	0.25	0.53	0.93	72.29
Triheptanoin	428.61	5.90	118.08	30	6	0	20	78.90	21070.65	0.03	0.76	0.68	0.95	82.75
Alanine	89.09	-0.58	21.29	6	3	3	1	63.32	2985.81	0.32	0.41	0.95	0.92	95.73
Acetic acid	60.05	0.09	13.31	4	2	1	0	37.30	2152.17	0.37	0.23	0.98	1.00	97.56
Tyrosine	181.19	0.35	47.42	13	4	4	3	83.55	3007.36	0.27	0.48	0.87	0.82	89.35
Lysine	146.19	-0.47	38.52	10	4	5	5	89.34	3943.08	0.24	0.53	0.70	0.99	93.82
Glutamic acid	147.13	-0.74	32.48	10	5	4	4	100.62	232.80	0.14	0.35	0.97	0.99	92.66
Blarcamesine	281.40	3.53	86.28	21	2	0	4	12.47	305.83	0.21	0.36	0.77	0.99	81.46
Dextromethorphan	271.40	3.38	81.37	20	2	0	1	12.47	2864.70	0.10	0.63	0.73	1.00	65.65
Vatiquinone	440.67	7.52	135.59	32	3	1	12	54.37	4139.20	0.24	0.11	0.65	0.87	75.77
Lovastatin	404.55	4.20	110.84	29	5	1	6	72.83	355.86	0.31	0.24	0.88	0.40	69.11
Desipramine	266.39	3.53	85.84	20	2	1	4	15.27	608.61	0.61	0.46	0.81	0.92	73.89
Sarizotan	348.42	4.37	100.52	26	3	1	5	34.15	3187.83	0.07	0.35	0.62	0.92	73.08
Cannabidiol	314.47	5.85	97.04	23	2	2	6	40.46	2625.60	0.48	0.21	0.85	0.85	79.89

Levodopa	197.19	0.05	49.09	14	5	5	3	103.78	6231.50	0.14	0.56	0.65	0.10	87.53
Gentamicin	477.60	-3.33	119.93	33	12	11	7	199.73	659.51	0.38	0.20	0.77	0.99	69.67
Fluoxetine	309.33	4.44	79.80	22	2	1	6	21.26	1469.90	0.54	0.05	0.72	0.98	82.46
Lamotrigine	256.10	2.01	64.11	16	5	4	1	90.71	421.56	0.37	0.58	0.68	0.97	85.69
Bromocriptine	654.61	3.19	164.16	43	10	3	5	118.21	327.17	0.25	0.80	0.84	0.43	40.53
Naltrexone	341.41	1.53	89.80	25	5	2	2	70.00	203.92	0.33	0.66	0.60	0.89	53.22
Mirtazapine	265.36	2.48	81.07	20	3	0	0	19.37	2591.38	0.35	0.55	0.68	0.21	69.26
Curcumin	368.39	3.37	102.02	27	6	2	8	93.06	1705.45	0.45	0.41	0.88	0.92	78.17
Trolox	250.29	2.49	67.31	18	4	2	1	66.76	33.03	0.16	0.30	0.72	1.00	77.00
Folic acid	441.40	-0.04	111.89	32	13	7	9	213.28	884.86	0.07	0.11	0.81	0.99	66.64
Memantine	179.31	2.69	54.32	13	1	2	0	26.02	800.36	0.16	0.50	0.69	1.00	62.45
Midazolam	325.77	4.32	89.05	23	3	0	1	30.18	5429.47	0.46	0.30	0.75	0.99	70.56
Benserazide	257.25	-1.76	61.48	18	8	8	5	148.07	698.58	0.27	0.98	0.65	0.45	85.47
Citalopram	324.40	3.81	90.91	24	3	0	5	36.26	1630.45	0.41	0.54	0.73	1.00	74.02
Tropoflavin	254.24	2.87	71.25	19	4	2	1	70.67	688.94	0.11	0.68	0.53	0.86	78.09
CX-546	247.29	2.08	67.19	18	4	0	1	38.77	14427.55	0.21	0.47	0.81	0.95	79.15
LM22A-4	339.35	-2.15	84.70	24	9	6	9	147.99	4320.92	0.14	0.88	0.65	0.67	83.66
Allopregnanolone	318.50	4.60	91.90	23	2	1	1	37.30	2188.48	0.03	0.14	0.98	0.99	64.28
2-propylpentanoic acid	144.21	2.29	40.94	10	2	1	5	37.30	1039.94	0.16	0.04	0.97	1.00	94.31
Diazepam	284.75	3.15	81.81	20	3	0	1	32.67	2283.36	0.08	0.20	0.93	1.00	77.70
Cannabidivarin	286.41	5.07	87.80	21	2	2	4	40.46	749.38	0.44	0.38	0.82	0.85	80.96
Cysteamine	77.15	-0.13	22.91	4	1	2	1	26.02	691.72	0.23	0.50	0.82	1.00	98.93

Tianeptine	436.96	4.19	114.05	29	6	2	8	86.71	3386.20	0.30	0.07	0.68	0.64	65.98
Vigabatrin	129.16	0.36	35.04	9	3	3	4	63.32	1250.27	0.29	0.32	0.82	0.94	94.17
Carbamazepine	236.27	3.39	73.53	18	3	2	0	46.33	602.91	0.21	0.82	0.54	0.66	74.68
Melatonin	232.28	1.86	67.24	17	4	2	4	54.12	214.60	0.28	0.61	0.90	0.75	78.11
Clonidine	230.10	2.36	60.39	14	3	2	1	36.42	482.50	0.43	0.41	0.87	0.93	87.46
Trazodone	371.87	2.36	104.17	26	6	0	5	45.78	494.05	0.20	0.61	0.54	0.96	73.66
Clenbuterol	277.20	3.00	73.59	17	3	4	3	58.28	23.93	0.67	0.35	0.87	0.97	85.63
Picrotoxinin	292.29	-0.06	66.83	21	6	1	1	85.36	27.34	0.66	0.58	0.51	1.00	50.84
Picrotin	310.30	-0.87	68.32	22	7	2	1	105.59	139.81	0.66	0.68	0.51	0.95	49.52

*The S^S average value of the reference set is 1.0.

Table S7. Values of Drug-Induced Liver Injury (DILI) of tacrine and its 10 best-scored derivatives.

**Derivative**	**admetSAR3.0**	**ADMETlab3.0**	**ProTox3.0**	**DILI-predictor**	**HTP**
TAC	Negative	Medium	Inactive	Positive	Positive
TAC-445	Positive	Poor	Inactive	Positive	Positive
TAC-572	Positive	Poor	Inactive	Positive	Positive
TAC-674	Positive	Poor	Inactive	Positive	Positive
TAC-678	Positive	Medium	Inactive	Positive	Positive
TAC-593	Positive	Poor	Inactive	Positive	Positive
TAC-798	Negative	Poor	Inactive	Positive	Positive
TAC-698	Positive	Poor	Inactive	Positive	Positive
TAC-485	Positive	Poor	Inactive	Positive	Positive
TAC-284	Positive	Poor	Inactive	Positive	Positive
TAC-175	Negative	Poor	Inactive	Positive	Positive

Table S8. First ionization energy (IE, eV) and bond dissociation energies (BDE, kcal mol^{-1}) for tacrine (TAC) and the selected subset of derivatives. The q represents the charge of the acid-base species.

Derivative	q	Structure	IE	BDE	BDE-site
TAC	1	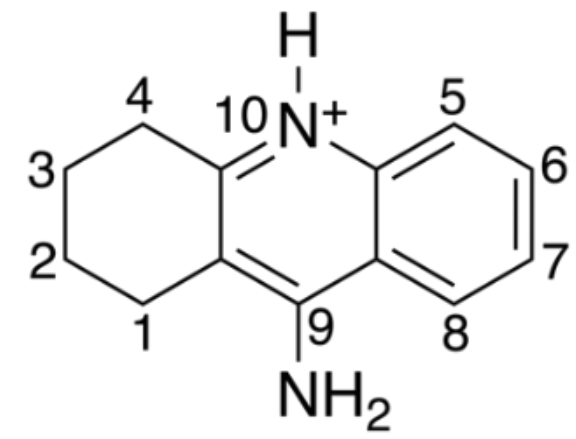	6.68	87.7	C4
				88.5	C1
				99.1	C2
				99.9	C3
	0	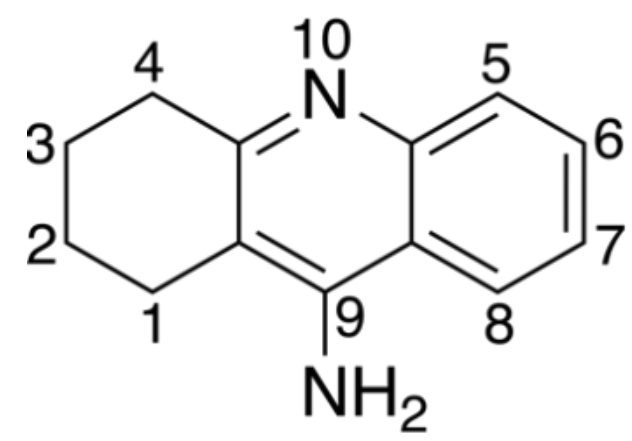	5.92	85.3	C1
				87.5	C4
				98.5	C2
				99.3	C3
TAC-678	1	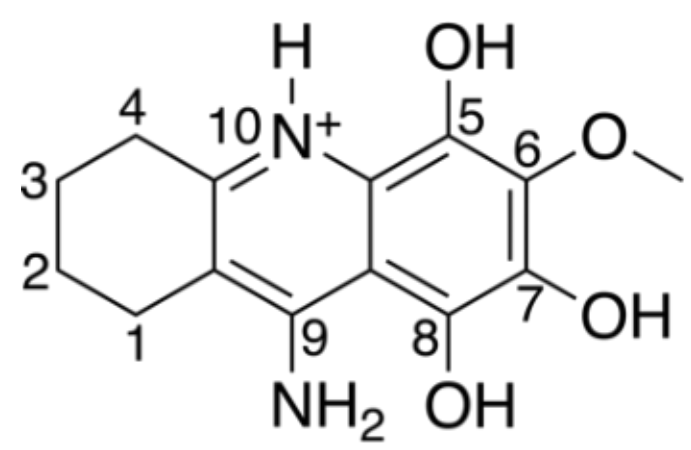	5.56	72.8	OH (C8, R_4)
				75.1	OH (C5, R_1)
				75.3	OH (C7, R_3)
				87.7	C4
				88.3	C1
				98.9	C2
				99.8	C3
				100.7	OCH_3 (C6, R_2)
	0	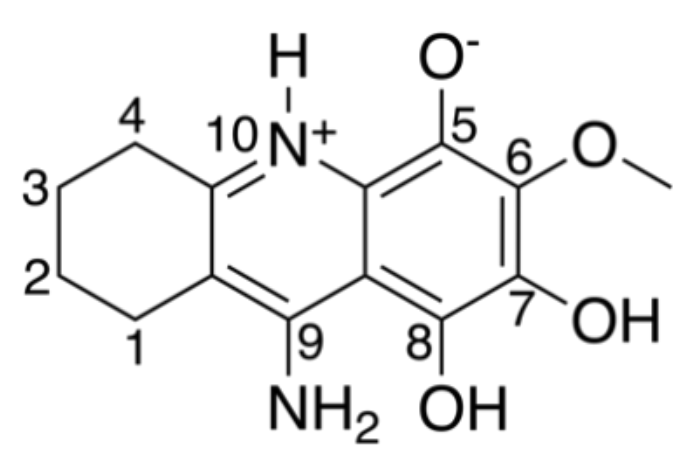	4.61	60.9	OH (C8, R_4)
				67.6	OH (C7, R_3)
				87.5	C4
				87.8	C1
				98.7	C2
				99.8	C3
				99.8	OCH_3 (C6, R_2)

	-1	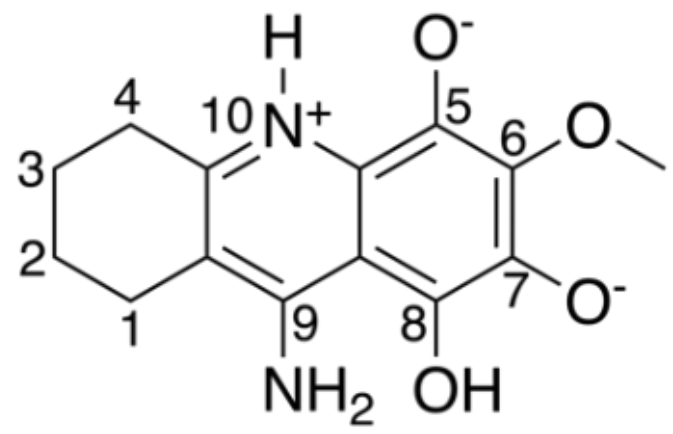	4.05	57.9	OH (C8, R_4)
				83.8	C4
				87.4	C1
				98.5	C2
				98.5	OCH_3 (C6, R_2)
				99.7	C3
TAC-674	1	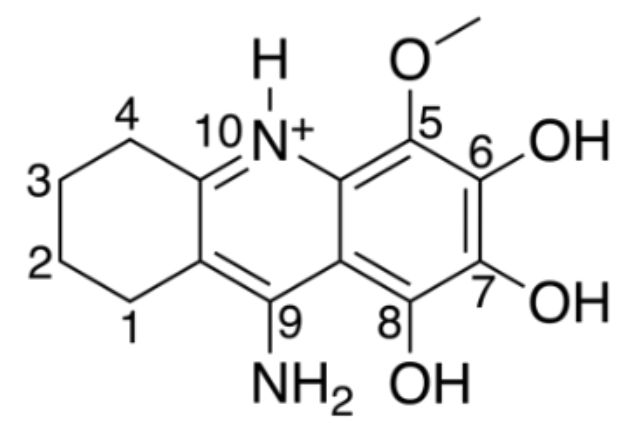	5.66	72.1	OH (C8, R_4)
				73.1	OH (C7, R_3)
				80.1	OH (C6, R_2)
				87.6	C4
				88.6	C1
				98.8	C2
				99.9	C3
				100.6	OCH_3 (C5, R_1)
	0	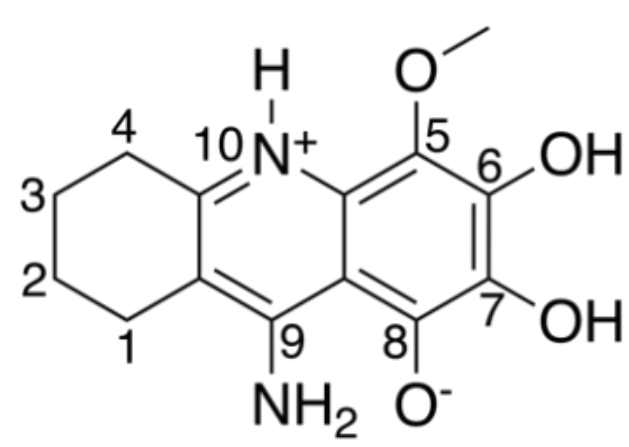	4.77	67.4	OH (C7, R_3)
				74.7	OH (C6, R_2)
				87.9	C4
				88.2	C1
				98.7	C2
				99.7	C3
				99.7	OCH_3 (C5, R_1)
	-1	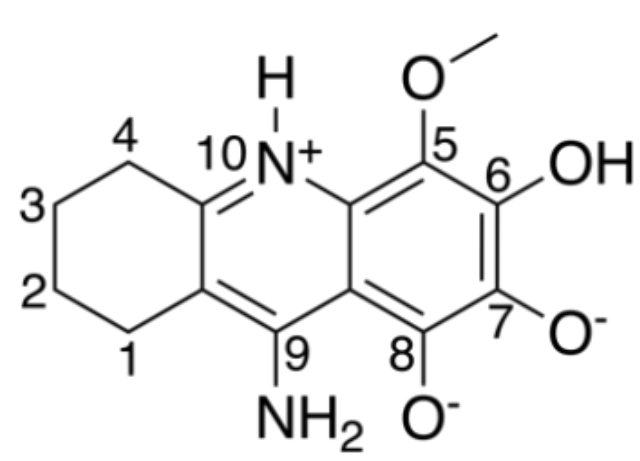	3.80	60.2	OH (C6, R_2)
				85.3	C4
				87.7	C1
				98.6	C2
				99.3	OCH_3 (C5, R_1)
				99.6	C3
$HOO^-/HOOH$			4.49	85.0	
Trolox			5.16	74.5	
Ascorbic acid			4.99	67.7	
α-Tocopherol			5.18	75.6	

Table S9. Binding energy (kcal mol^{-1}) and weighted binding energy (kcal mol^{-1}) of the acid-base species for tacrine and the selected subset of derivatives.

**Derivative**	ΔG_B			ΔG_B^W
	q **= 1**	q **= 0**	q **= -1**	
		**AChE**		
TAC	-8.7			-8.7
TAC-678	-8.6	-8.8	-9.4	-8.7
TAC-674	-9.4	-9.7	-9.8	-9.7
		**NMDAr**		
TAC	-6.6			-6.6
TAC-678	-6.9	-7.1	-7.3	-7.0
TAC-674	-6.9	-7.0	-7.2	-7.0
		**COMT**		
TAC	-5.5			-5.5
TAC-678	-5.6	-5.7	-5.7	-5.7
TAC-674	-6.2	-6.1	-5.7	-6.1
		**MAO-B**		
TAC	-9.3			-9.3
TAC-678	-8.9	-8.8	-8.8	-8.9
TAC-674	-8.2	-8.6	-8.7	-8.6

Table S10. Interactions of tacrine and the subset of selected derivatives with the proteins used to study the interaction pattern.

Compound	AChE	NMDAr	COMT	MAO-B
TAC	Trp286(πs, πA), Tyr341(πs, πA)	Val644C(A), Ala645C(A), Val640D(πA), Leu643D(πσ, A), Ala644D(πA)	Met40(A), Pro174(πA), Glu199(H)	Leu171(πA,A), Cys172(πS,A), Tyr326(πA), Tyr398(πs)
TAC-674	Trp86(H, πs), Asn87(UH), Tyr124(H), Tyr337(πA), Phe339(πA), Tyr341(πA)	Thr648C(UD,H), Val640D(A), Leu643D(A), Ala644D(A), Thr647D(UH)	Met40(πA), Lys144(H), Asn170(UH,UA), Pro174(πs), Mg(CB)	Leu171(πA, A) Cys172(A), Ile199(A), Tyr398(πS), FAD(H, UH)
TAC-678	Asp74(H), Trp86(πS, UH), Tyr124(πT), Tyr133(UH), Tyr337(πA), Phe338(πA), Tyr341(πA)	Asn615B(H), Val640B(UH), Val644C(πA), Thr648C(UD), Asn615D(H), Val640D(A), Leu643D(πA, A), Ala644D(A), Thr647D(H)	Trp143(πS, πA), Lys144(H), Asp145(H)	Leu164(A), Leu171(πσ), Cys172(H), Ile198(H, UH), Ile199(H, πσ, πA,A), Gln206(H), Ile316(A), Tyr326(H)
NS	Tyr72(πC+, UH), Trp86(πC+), Tyr124(H), Tyr133(UH), Trp286(πσ, πC+), Tyr337(UH), Tyr341(πσ)	Ser505B(H), Gly672(UH), Ser673B(H), Asp715B(H)	Trp38(UH, πT), Met40(πA), Asn170(H), Pro174(πA), Glu199(H), Mg(CB)	Ile199(H), Tyr398(πs), Tyr435(H), FAD(H)
RD	Tyr72(UH), Trp86(πs), Trp286(πs, πσ, πA), Ser293(UH), Phe295(H), Tyr337(πA), Phe338(πA), Tyr341(πs, πA, πσ)	Asn615B(H), Val644C(A), Leu643D(A), Ala644D(A)	Met40(πA), Asp141(H), Lys144(H), Asn170(H), Pro174(πA), Glu199(H), Mg(CB)	Leu171(πσ), Cys172(πS), Ile199(πA, UH), Gln206(H, UH), Ile316(πA), Tyr326(πS, UH), Tyr343(πA), Tyr398(πA), FAD(πLP, πA)

NS: Natural Substrate, RD: Reference Drug, Interaction type: H: Conventional hydrogen bond, UH: Unconventional hydrogen bond, πA: π-alkyl, πs: π-π stacking, πT: π-π T-shaped, πσ: π-σ interaction, πS: π-Sulfur, πC^+: π-cation, πLP: π-lone pair, A: alkyl, CB: coordination bond, D: donor-acceptor, UD: unfavorable donor-donor, UA: unfavorable acceptor-acceptor.

Table S11. Interaction-matching parameters used for the calculation.

Protein	Candidate	Reference	NRR	NMR	NMI	S_{SI}
AChE	TAC	NS	7	2	0	0.14
AChE	TAC	RD	8	2	2	0.25
AChE	TAC-678	NS	7	5	1	0.43
AChE	TAC-678	RD	8	4	4	0.50
AChE	TAC-674	NS	7	4	1	0.36
AChE	TAC-674	RD	8	4	4	0.50
NMDAr	TAC	RD	4	3	2	0.63
NMDAr	TAC-678	RD	4	4	3	0.88
NMDAr	TAC-674	RD	4	2	2	0.50
COMT	TAC	NS	6	3	2	0.42
COMT	TAC	RD	7	3	2	0.36
COMT	TAC-678	NS	6	0	0	0.00
COMT	TAC-678	RD	7	1	1	0.14
COMT	TAC-674	NS	6	4	2	0.50
COMT	TAC-674	RD	7	5	3	0.57
MAO-B	TAC	NS	4	2	1	0.38
MAO-B	TAC	RD	9	4	1	0.28
MAO-B	TAC-678	NS	4	2	1	0.38
MAO-B	TAC-678	RD	9	6	3	0.50
MAO-B	TAC-674	NS	4	3	1	0.50
MAO-B	TAC-674	RD	9	5	0	0.28

NS: Natural Substrate, RD: Reference Drug.

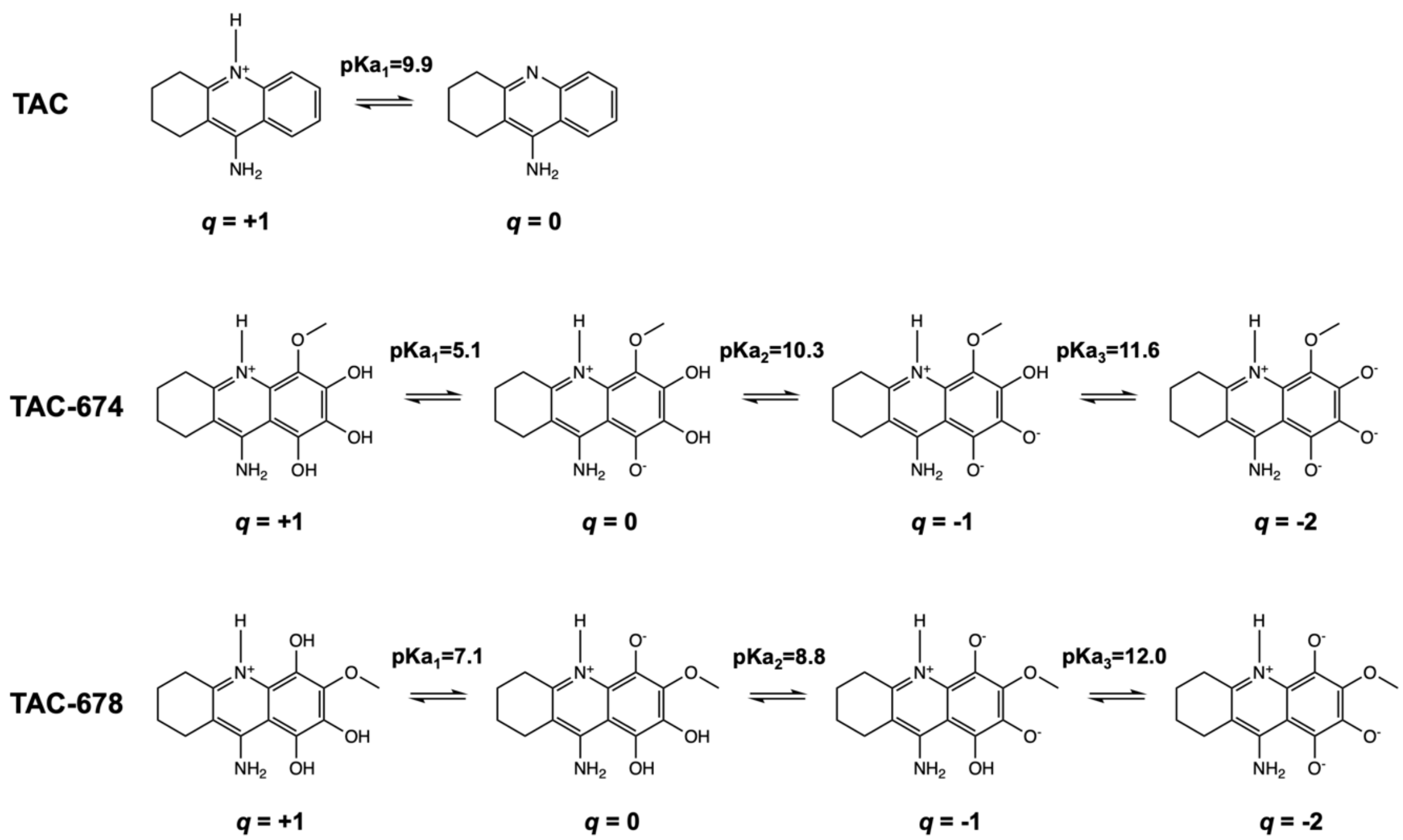

Figure S1. Deprotonation routes for tacrine and the derivatives selected as the most promising, due to their drug-like behavior.

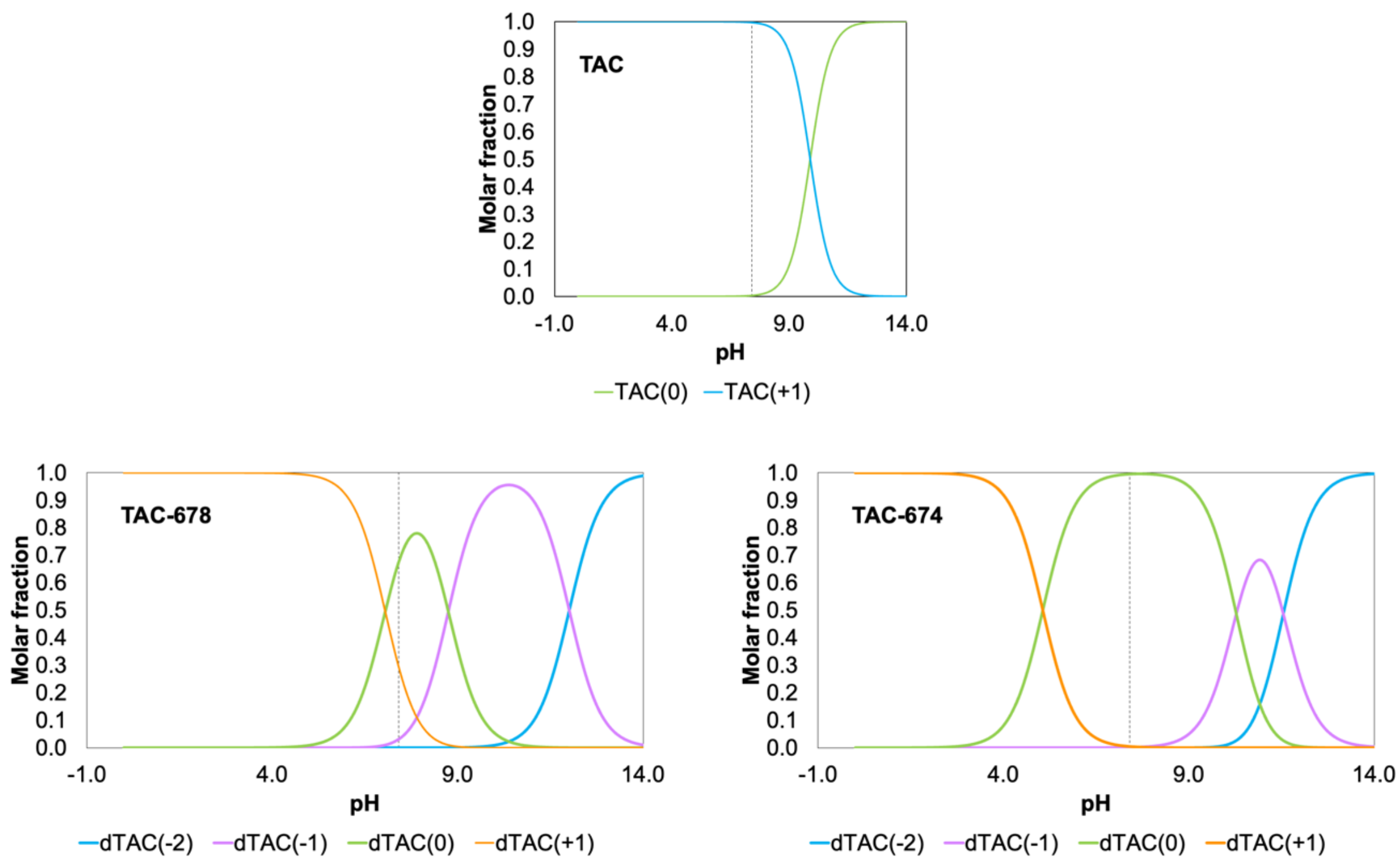

Figure S2. Molar fractions for tacrine and the subset of derivatives selected as the most promising, due to their drug-like behavior. The vertical dotted line corresponds to the molar fractions at pH = 7.4.

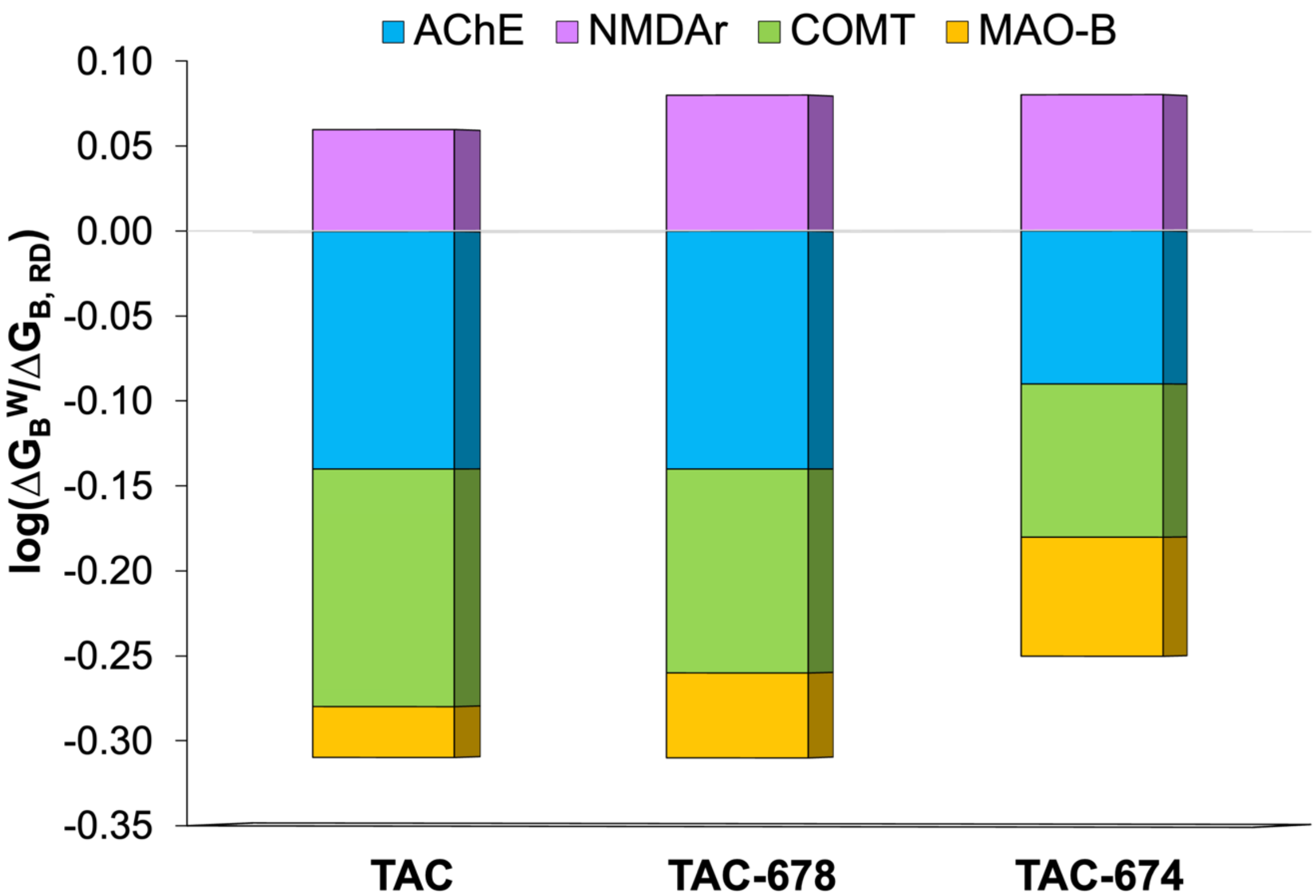

Figure S3. Multi-target efficiency plot of tacrine and its derivatives relative to the reference drugs.

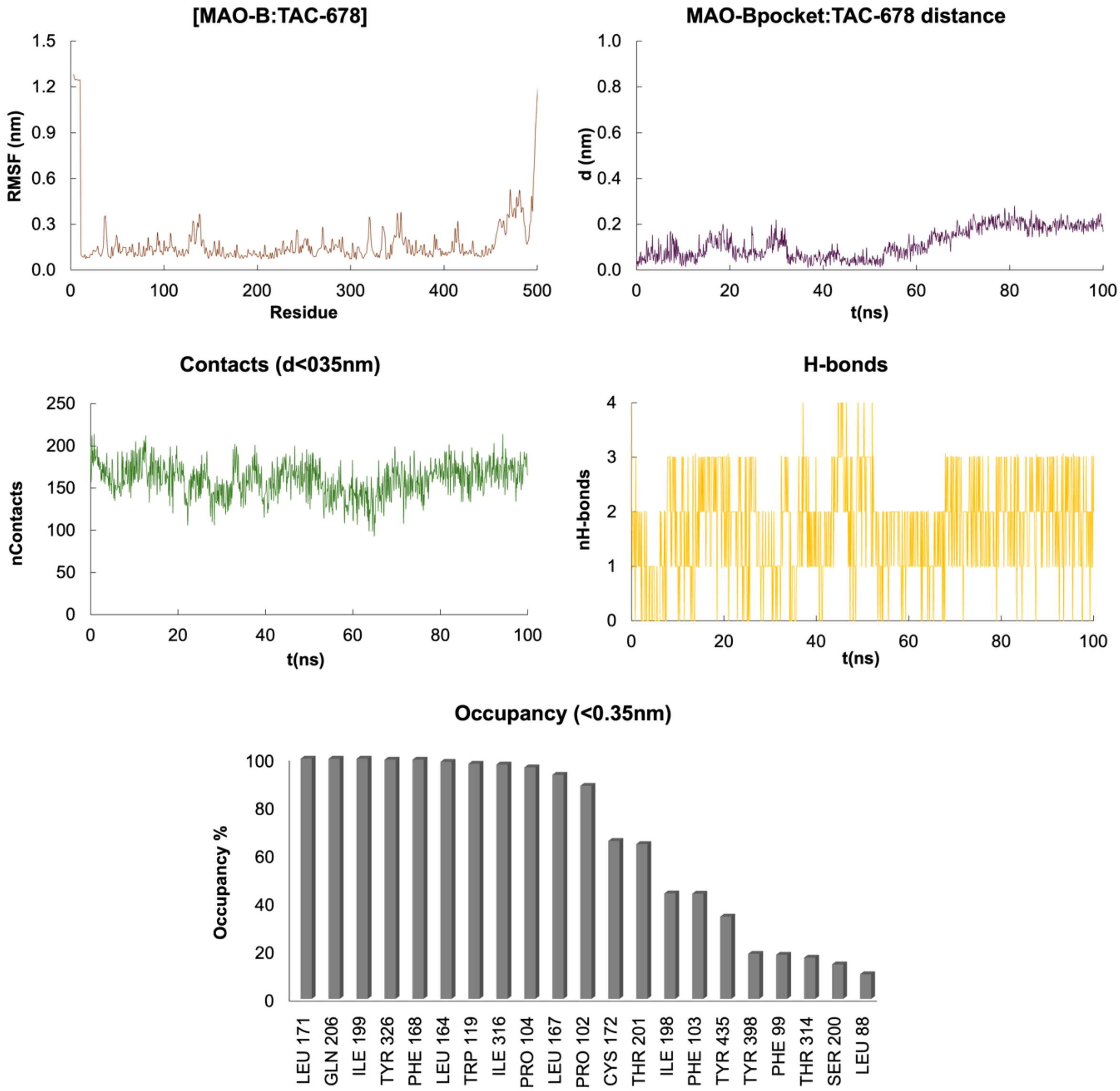

Figure S4. MD-100ns plots for the complex [MAO-B:TAC-678] complex.

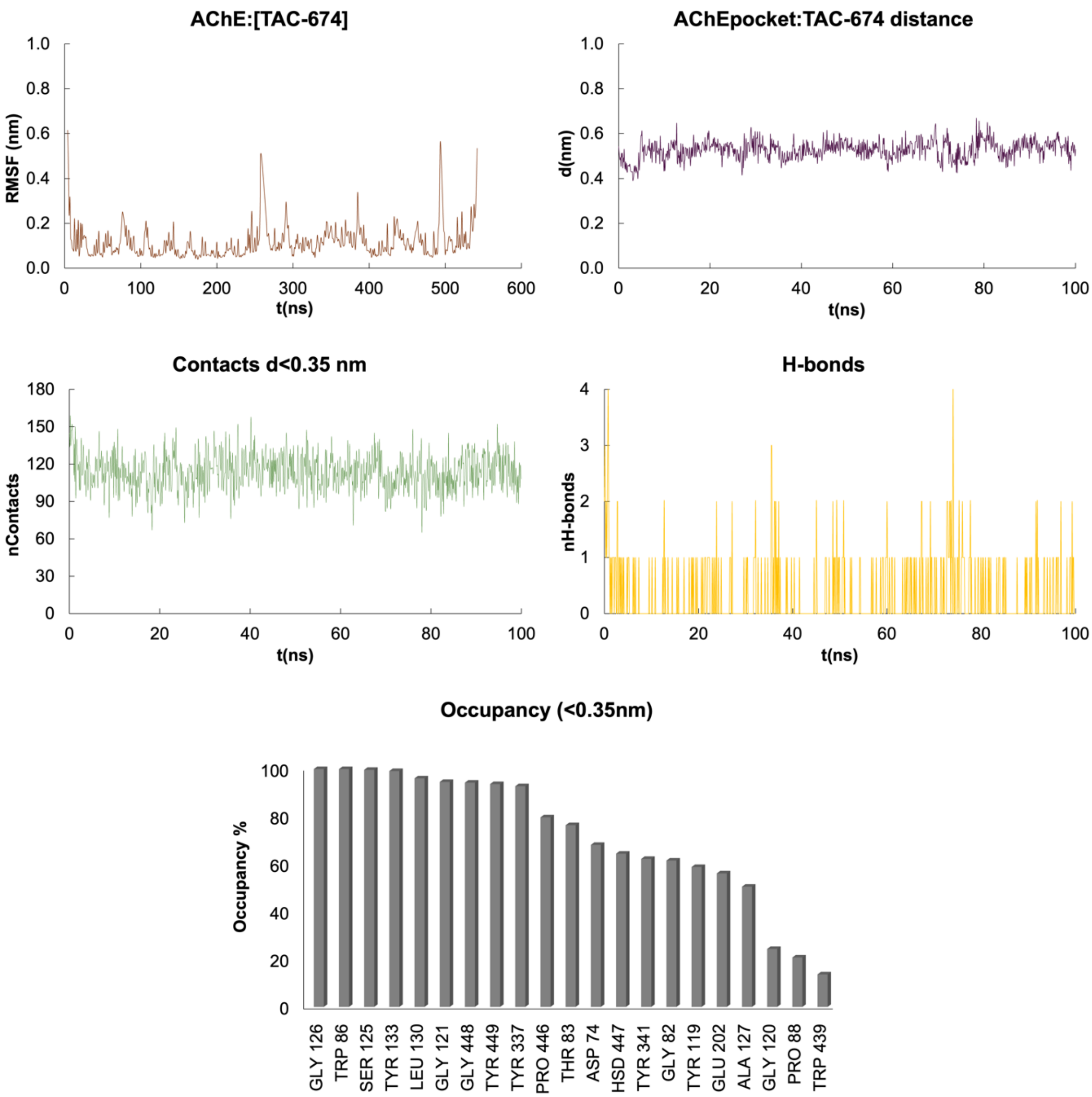

Figure S5. MD-100ns plots for the complex [AChE:TAC-674] complex.
